# Co-design of a digital 24-hour time-use intervention with older adults and allied health professionals

**DOI:** 10.3389/fdgth.2025.1544489

**Published:** 2025-05-23

**Authors:** Henry T. Blake, Aaron Davis, Maddison L. Mellow, Melissa Hull, Bethany Robins, Kate Laver, Dorothea Dumuid, Timothy Olds, Hannah A. D. Keage, Lui Di Venuto, Ashleigh E. Smith

**Affiliations:** ^1^ Alliance for Research in Exercise, Nutrition and Activity (ARENA) Research Centre, Allied Health and Human Performance, University of South Australia, Adelaide, SA, Australia; ^2^ Australian Research Centre for Interactive and Virtual Environments (IVE), UniSA Creative, University of South Australia, Adelaide, SA, Australia; ^3^ Division of Rehabilitation, Aged and Palliative Care Service, Southern Adelaide Local Health Network, Bedford Park, SA, Australia; ^4^ Caring Futures Institute, College of Nursing and Health Sciences, Flinders University, Bedford Park, SA, Australia; ^5^ Justice and Society, University of South Australia, Adelaide, SA, Australia; ^6^ City of Onkaparinga, Adelaide, SA, Australia

**Keywords:** co-creation, physical activity, sedentary behavior, sleep, 24-hour activity cycle, intervention, health promotion

## Abstract

Using co-design principles based on the Health CASCADE framework, we aimed to describe the collaborative process undertaken to develop a 24-hour time-use intervention, called Small Steps, which promoted gradual and incremental health-behavior change. A secondary aim was to reflect on the challenges and benefits of co-design in this project, offering insights into the “why” and “how” to co-design 24-hour time-use interventions with priority populations. Twelve participants were invited and participated in 6 co-design workshops (June 2023–January 2024). To prioritize older adults’ views in the end-product, 8 adults aged >65 years (the target population) and 4 allied health professionals with >2 years’ experience working with the target population were recruited. Workshops and activities were structured using the British Design Council's Double Diamond Design Process to stimulate design thinking. Where possible, participant-led documentation was used to reduce the bias associated with academic scribing and empower participants to provide input and facilitate ownership for the project. Workshop activities and discussions were captured through printouts, audio and iPad screen recordings and analyzed through reflexive thematic and content analysis. Co-designers contributed to all elements of the intervention including the website design, the content, and the level of researcher input during the intervention. Iterative improvements were made based on the unique perspectives and needs of the community experts. During the action planning process, older adults wanted both support and autonomy, while maintaining the freedom to adapt these options to their individual needs. Older adults also preferred a step-by-step approach, allowing for gradual behavior changes across the intervention to avoid feelings of becoming overwhelmed. The co-design process enabled the tailoring of the Small Steps intervention to the specific needs of its intended audience. Key factors contributing to the co-design included flexibility in the design process, fostering a supportive environment, and empowering participants through activities that guided and stimulated their thinking. These elements not only helped shape the development of Small Steps but reinforced the value of co-design in developing personalised interventions for older adults.

## Introduction

There is a growing recognition that daily behaviors including physical activity (PA), sedentary behavior (SB), and sleep are interrelated and collectively influence cognition in older adults (1–3). These behaviors make up the 24-hour activity cycle, where increasing time spent in one activity domain, for example PA, is compensated by an equal and opposite net change in the other activity domains (i.e., SB or sleep). This integrated research approach is relatively new, made possible by the increasing availability of 24-hour wearable devices (e.g., accelerometers) and new analytical methods to enable the exploration of all daily activities simultaneously (e.g., compositional data analysis) (4, 5). Previously, interventions only targeted these behaviors in isolation, for example, increasing PA, reducing SB or targeting aspects of sleep in an isolated manner, with no consideration of which behavioral swaps were being made (6). However, because daily activities are mutually exclusive and exhaustive, understanding how time is reallocated, and which changes are more feasible or sustainable, may be critical for improving health outcomes including cognition (7, 8). Despite this potential, no interventions have applied a 24-hour time-use framework to cognitive health, nor involved older adults in their design using co-design (9). This study directly addresses these gaps.

**Figure 1:**
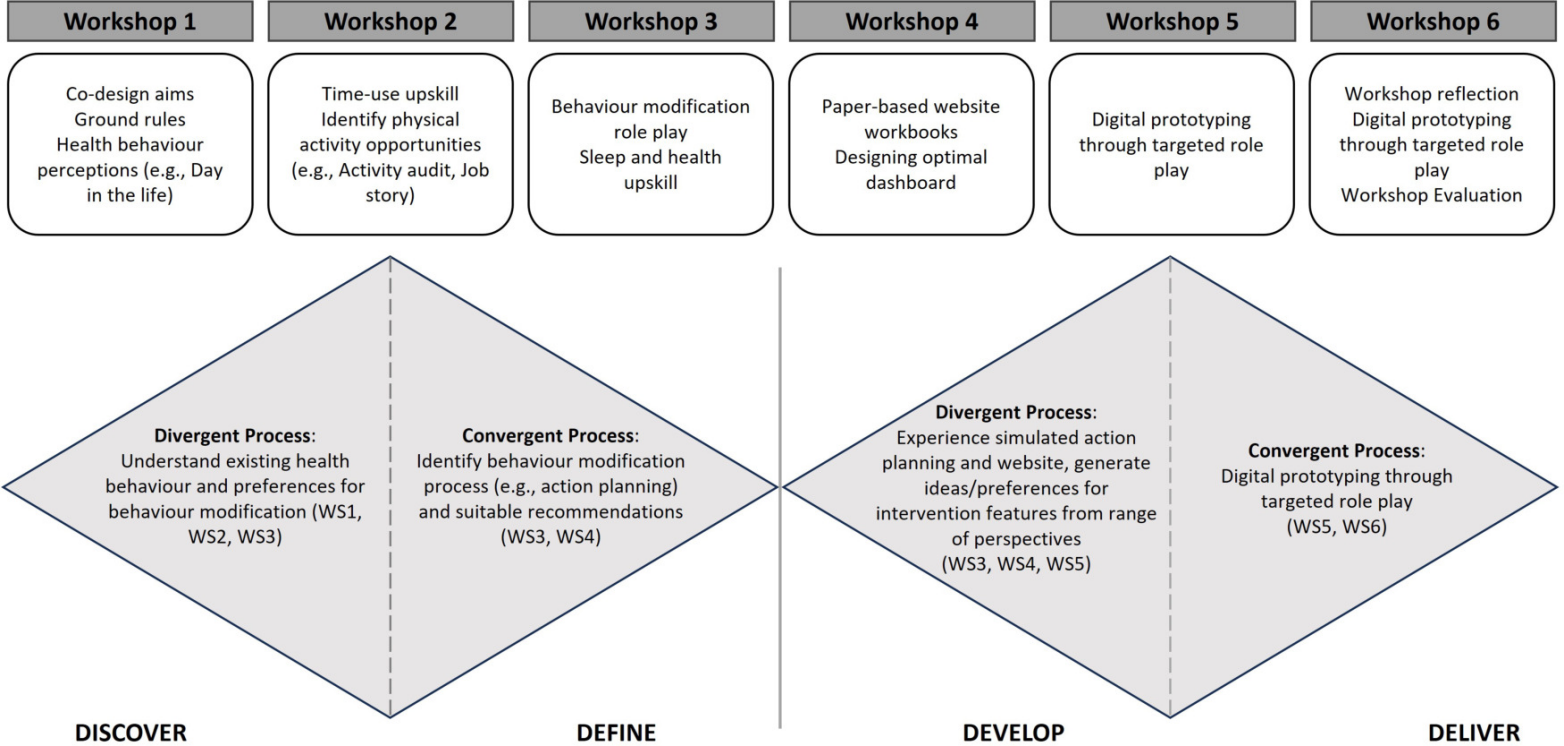
Workshop overview and mapping the intention of activities to the Double Diamond approach. WS, Workshop.

Participation in healthy behaviors is influenced by various personal and intervention design factors. Social disadvantages contribute to health inequalities and are crucial, yet often overlooked in 24-hour activity cycle and cognitive health research (10). A recent study incorporating data from nearly 2 million participants representing 96% of the global population revealed almost a quarter of all adults do not get enough PA (11). Low socioeconomic status is a common correlate of poorer adherence to PA interventions across populations, including older adults with and without dementia, and is linked to sleep disturbances among older adults which can further impact cognition (12–14). Participants from lower socioeconomic households may have unique time constraints, knowledge, and barriers to adhering to PA and time-use interventions, and a one-size-fits-all intervention is not likely to be appropriate.

Technology-based interventions, such as internet applications, show promise in empowering older adults to adopt physical activity behaviors (15). These interventions are becoming more accessible, including for people living with cognitive impairment and dementia. For instance, one study reported that 90% of older adults used email daily, and over 80% indicated they would use an eHealth intervention (16). The rapid adoption of digital technology since the start of the COVID-19 pandemic has further accelerated this trend (17). Despite this progress, many older adults continue to value trusting relationships in behavior change, underscoring the importance of maintaining human contact (15). Research supports this, demonstrating that combining web-based interventions with human support yields greater improvements in cardiovascular risk factors, including PA, among middle-aged and older adults (18). While the accessibility, adaptability and functionality of web-based interventions are promising, they require thoughtful planning and consideration. Therefore, to optimize the success and sustainability of digital 24-hour interventions targeted at older adults, working with the target population is essential.

Co-creation is an established methodology for actively engaging end-users and key stakeholders together in intervention design (19), and shows promise in improving the effectiveness and sustainability of health interventions in a specific context (19, 20). Co-creation involves the public in research and ensures interventions are best tailored to meet the needs of the end users. To better align with the Australian health research landscape where this research was conducted, co-design terminology will be used throughout the remainder of the manuscript. “Co-design” is a term more commonly used in Australia, but the aims and research methodologies in the context of this study closely overlap with co-creation. One potential criticism of co-design is that its flexibility and adaptive nature presents a challenge for reproducibility and scientific rigor (21). To overcome these issues in reproducibility and rigor, the European-funded 2020 Marie Sklodowska-Curie Innovative Training Network (ITN) Health CASCADE project developed an open-source framework for co-design upon which this project has been constructed (21).

We aimed to describe the collaborative co-design process undertaken with older adults and health-care professionals in a lower socioeconomic area in outer metropolitan and inner regional areas of Adelaide, South Australia to develop a web-based, telephone-supported, 24-hour time-use intervention. A secondary aim of this study was to reflect on the challenges and benefits of co-design in this project and provide insight into its effectiveness for this study. The resultant Small Steps intervention aims to help older adults improve their daily time use for cognitive health. Co-designers contributed to all aspects of intervention development including the creation of a digital mobile-friendly website, determining the essential components of the interface, organizing information flow throughout the intervention, and identifying the necessary level of detail for the digital tool. Here, we present an outline of the chosen methods, with description of the choices made, and reflection on the process. We expect a forthcoming protocol paper will outline the final intervention and delivery protocol for the trial.

## Methods

### Overview of Small Steps funding and researcher reflexivity statement

The “Small Steps towards personalised dementia prevention” project was funded by a Medical Research Future Fund (MRFF) Effective Treatments and Therapies grant (MRF2022954). This grant aimed to co-design, implement, and evaluate a personalised technology-assisted 24-hour time-use intervention to promote positive behavior change, such as increased PA or improved sleep quality, and reduce modifiable lifestyle dementia risk factors. The senior author (AS) led the grant application and the research team. The research team (authors) included multidisciplinary researchers with a predominant focus on time use and PA for health across the lifespan (HB, MH, MM, DD, TO, and AS), cognitive ageing (HK, AS, MM), co-design (AD), implementation science (KL), and industry partners (LD).

### Overview of co-design workshops

Six two-hour co-design workshops were held over a period of 6 months with older adults and allied health professionals to capture their views and preferences for the development of a web-based 24-hour time-use intervention (July 2023–January 2024). The first author (HB) and second author (AD), an academic with extensive co-design experience, facilitated the workshops. Where needed, additional academics from the research team and software developers also attended and participated in the workshops (see Supplementary Material).

The research was approved by the University of South Australia Human Research Ethics Committee (no. 205377) and all procedures were conducted in accordance with the Declaration of Helsinki.

This study employed a participatory co-design methodology, with stakeholders (older adults, health professionals, researchers and IT developers) engaged as equal contributors in the development of the intervention. The study design was informed by several traditional qualitative methodologies including Yin's case study methodology (22) to situate the work within the real world, and employed analytical processes from grounded theory (23), including constant comparison to support interpretation of the data.

### Stage 1: workshop planning

The co-design process was framed using the PRODUCES framework (Table 1) from Health CASCADE (25). Each of the workshops were structured using the British Design Council's Double Diamond (24) design process (Discover, Define, Develop and Deliver) (Figure 1):

• **Discover:** Understand older adults’ experiences of and attitudes towards healthy behaviors, specifically increasing their PA or improving sleep, and identify preferences regarding behavior change
• **Define:** Identify the desired features of a 24-hour time-use intervention that could be implemented in this population
• **Develop:** Develop the intervention and the interface considering a range of perspectives and inspirations
• **Deliver:** Test multiple versions of the prototype and refine the product with iterative feedback

**Table 1:** Framing the workshops using PRODUCES framework.

PRODUCES	Explanation	Application
Problem	What is the reason for the process?	Many older adults are insufficiently active and experience disturbed sleep. A change in PA comes at the expense of either sitting time or sleep, and vice versa. Previous behavioral interventions have not taken this into consideration.
Objective	What is the aim of the process?	To work collaboratively with community members to gain insight into their lived experience and design a personalised intervention to help older adults to achieve the best make-up of their 24-hour day for cognitive health.
Design	What specific participatory methodology is used for co-creation?	Double Diamond Design process (24) were used to guide and plan the workshop activities.
(End-) users	Who will use the co-created intervention?	(1) Ambulatory and community-dwelling older adults (65 years of age and older) living within the local council region where the intervention will be later tested. (2) Allied Health professionals who work closely with older adults in a health promoting capacity, e.g., exercise physiologists, personal trainers, physiotherapists, or occupational therapists. Other health professionals who work in a health promotion capacity with older adults (e.g., care workers, community health promoters).
Co-creators	Who is engaging in the process?	University researchers, community-dwelling older adults, community-based exercise physiologists and exercise professionals, and website developers.
Evaluation	How is success measured?	(1) Post-workshop semi-structured interviews investigating participant experience and perception of final co-designed product representing participant input (results presented elsewhere) (2) Embedding the co-designed intervention into a randomised controlled trial within the focus community.
Scalability	How can the solution be scaled to a population level?	Cascade model (design of a local intervention and local implementation before being transported/adapted to a new group in a different setting).

Multiple strategies were utilized throughout the study to mitigate potential power imbalances between co-designers. Firstly, participant-led documentation was used where possible to reduce the bias associated with academic scribing and empower each participant to provide input into the project. Where older adult co-designers requested, a member of the research team or a health professional took notes on their behalf, but with structured member checking to ensure written data accurately captured participant perspectives. In addition, workshop activities and discussions were also captured through audio and iPad screen capture recordings.

### Participant recruitment and screening

To facilitate high-quality interactions among co-designers and ensure diverse participant perspectives, 10–12 participants fluent in English were recruited using purposive convenience sampling. Sampling focused on balancing genders and including both older adults and health professionals who may be involved in participating in or administering such a program in the future. The older adult and health professional sample are referred to as “co-designers” throughout this manuscript. Prior to participation all participants provided informed written consent. All participants received an honorarium for each workshop in recognition of their time.

Older adult co-designers (≥65 years) were recruited through electronic and paper advertisements via ACH Group and City of Onkaparinga networks, along with a targeted social media advertisement. Exclusion criteria for older adults included a score below the mild cognitive impairment cut-off (<13/22) on the Telephone Montreal Cognitive Assessment (T-MoCA) (26), current dementia diagnosis, major neurological or psychiatric diagnosis, known intellectual disability, major physical disability, inability to attend most workshops, or residence outside the City of Onkaparinga. Eligible older adults attended a one-on-one session at a local council facility with the first author (HB), where demographic information and health information was obtained through the completion of the CogDrisk (27, 28). If older adults requested to attend the co-design workshops with a care giver, family member or intermediary, this was honored.

Health professional co-designers were recruited from the ACH Group and by word-of-mouth. Eligibility screening was conducted through an online questionnaire and brief phone interview to ensure understanding of study requirements and comfort with the collaborative approach. Exclusion criteria for health professionals included less than 2 years of experience working with older adults in an exercise capacity, not regularly working with older adults, or unable to attend most workshops. Importantly, health professionals participated as equal co-designers, contributing both clinical expertise and tacit knowledge from working with older adults. To build empathy and deepen their understanding of the intervention, all co-designers completed 7-days of habitual 24-hour time use monitoring using a wrist-worn triaxial accelerometer (Axivity AX3, Axivity Ltd, Newcastle, UK). This activity was intended to prompt reflection and discussion on the practicalities of the intervention's tools and strategies.

### Stage 2: workshop format and content

Workshops were held in a large room within community centers that were local, familiar, and comfortable for the older adult co-designers, as recommended (29). To promote socialization and positive group dynamics, in-person workshops were planned and most activities were completed in small groups ensuring opportunities for equal contribution (20). At the start of each workshop, co-designers were introduced to the facilitators, presented a recap of the previous workshop, upskilled on key content relevant for the workshop, and informed on the respective workshop objectives and plan. Co-designers then engaged in a series of interactive activities. Each workshop included an ice-breaker activity aimed at building rapport among co-designers and to stimulate creative thinking. While a semi-structured outline for all workshops was developed prior to Workshop 1 (available within the Supplementary File), workshop plans and activities were iteratively refined throughout with the needs of the co-designers considered.

### Workshop 1: discover

Workshop 1 aimed to discover the co-designers' understanding of healthy behaviors and foster a sense of ownership over the project through upskilling and generation of group rules. The lead facilitators (HB and AD) explained the purpose of co-design, its application to the current project, and presented current scientific understanding of PA, SB, and sleep and their relationship with cognitive health. Initial ownership was promoted through defining co-designers' roles, with older adults identified as “community experts” and allied health staff as “health professionals”. Co-designers were given the opportunity to design their own study name tags to reflect their preferred names and a unique skill they possess, followed by discussion among co-designers to get to know one another. To gauge their understanding of a healthy 24-hour day, they individually created “day in the life” diagrams, illustrating what they believed constituted healthy and unhealthy days (health professionals embodied an older adult when completing the activity). A custom activity sheet was then used for them to categorize behaviors as “healthy”, “unhealthy”, or “unsure/both”. The overall results were compiled and discussed at each table.

Facilitators further encouraged a sense of ownership by promoting openness and equal contribution from all co-designers. Individually, in small groups, and then as a whole group, ground rules for the workshops were developed. Between Workshops 1 and 2, the lead facilitator (HB) compiled the group responses into five rules of engagement, and verified these with the co-facilitator (AD), before presenting these at the start of Workshop 2. A gradient of agreement exercise ensured all co-designers were satisfied with the compiled rules.

### Workshop 2: discover and define

Workshop 2 focused on using generative techniques to further explore co-designers' existing health behavior knowledge, to define PA opportunities, and examine behavior planning for older adults.

To identify suitable PA opportunities for older adults that might form the basis of the intervention, an “Activity Audit”, open-ended brainstorming, and “Job Story” activities were completed. In brief, co-designers first audited a list of activities developed from a PA compendium (30) to determine whether an activity was appropriate for them, for any older adult, or if they considered the activity listed to be inappropriate for an older adult.

Open-ended brainstorming then guided co-designers to recognize common SBs of an older adult and identify alternative PAs. They then completed a “Job Story” exercise independently, identifying a modifiable SB that could be replaced, and progressing through three response options of increasing detail. These options ranged from simply selecting a replacement PA to specifying details like location, companions, duration, and additional open-ended factors. At each table, co-designers reflected on their preferred level of detail to promote behavior change and adherence to the choice. The workshop concluded with co-designers anonymously writing down what questions they may have if they were enrolled in the intervention in the future.

### Workshop 3: discover and define

Between Workshops 2 and 3, the researchers extended the “Activity Audit” and “Job Story” exercises by developing a paper-based action planning prototype informed by earlier workshops. This prototype was presented as a folded paper prototype, which unfolded to reveal each action planning step sequentially with pre-set options, simulating a potential future digital interface. Small groups of two older adults and one health professional role-played a behavior modification scenario using the prototype, and a hypothetical 24-hour day profile vignette. The older adults were encouraged and supported by the health professionals, to use the paper-based prototype to identify what SB could be replaced, with what PA, and then “where/when”, “how often”, “how long”, “with whom” the PA behavior could be implemented, and “what else do I need”. The activity was then repeated with a focus on modifying sleep.

Prior to the sleep task, co-designers were upskilled on the importance of sleep duration and quality for cognitive health, covering topics of sleep hygiene and stimulus-control strategies (31). Using a sleep focused paper-based prototype and 24-hour day vignettes, co-designers identified a behavior associated with poor sleep, such as watching TV in the bedroom, and identified an alternate sleep hygiene or stimulus-control behavior which could be used as a replacement. Considerations of the frequency and additional supports required for the behavior change were probed (i.e., an alarm, a book to read).

Throughout the paper-based action planning, health professionals prompted discussions at tables using semi-structured questions around co-designers' experiences. Discussions were centered around what was, and was not useful for the role play scenario or themselves, and the suitability of the options provided or perspectives on alternate options that older adults would have liked to see. Further, information on beliefs, flow of the action planning activity, and the level of detail of the action planning process were sought.

At this point in the workshops, the web-developer was starting to make decisions about the look and feel of the web platform. In response to this, the workshop was concluded with individual consideration and then table based discussion about what makes information “simple and easy to understand” or “complex and hard to understand”.

### Workshop 4: define and develop

Between Workshops 3 and 4, the research team developed a paper-based printout of the intended website design, incorporating the weekly action planning elements and perspectives on clear communication gathered from previous workshops. The initial paper-based website proposed visual and functional design elements for review. Co-designers worked individually through small workbooks featuring a still image of the website element on one page, with the opposing page free for co-designer-led documentation regarding both positive aspects and suggestions for improvement. The final workbook page asked for written responses to the following question: “after making your new selection for the week, what would you expect the next screen to be?”.

Digital interventions offer a customizable platform to meet users' needs and promote participant engagement (32). To leverage this, co-designers were asked to individually design their ideal website dashboard for the proposed intervention, in terms of features and content. A template and stickers of pictures/symbols were provided to promote thinking about what features should be included (i.e., educational resources). Dashboards were interpreted and collated by HB.

### Workshop 5: develop and deliver

Iterative changes to the digital prototype had been made based on recommendations identified by the co-designers, including desired website features between Workshops 4 and 5. Workshop 5 was focused on critiquing and enhancing the user experience with digital prototypes of the developing intervention.

Activities involved small group role play, whereby two older adults worked with a health professional or workshop facilitator to navigate several instructional scenarios embodying a 68-year-old man. The co-designers interacted with touch-screen tablets and the first digital prototype of the intervention website. Their experience and insight were collected through the think-aloud methodology (voice recording) and screen recording of the iPads was captured simultaneously to enable later cross-reference (33, 34). Health professionals were also prompted to manually write comments relating to their groups experience. The small groups followed instructional sheets that guided them to explore all aspects of the potential website features, and these instructional sheets progressed in the level of autonomy required by co-designers.

### Workshop 6: develop and deliver

The website underwent further iterative improvements based on co-designer feedback from Workshop 5. Workshop 6 began with a presentation summarizing the previous five workshops' progress and contribution to the final prototype, emphasizing co-designers' contribution. Prototype testing continued in the same format as Workshop 5, with co-designers using role play (new scenarios), instruction sheets (increasing in autonomy), and iPads. Feedback and experience was captured through the think-aloud methodology (audio recording) and simultaneous screen recording (33, 34). Activity sheets were designed to have the small groups navigate selected website/intervention elements, and to experience modifications made based on the previous workshop feedback.

Towards the conclusion of Workshop 6, co-designers enjoyed a light lunch and socialised before a final discussion reflecting on the process. All co-designers were invited to schedule a semi-structured phone interview with an independent researcher (MH) to gain insights into their co-design experience, as recommended (25). Examining co-designer experiences in PA research with older adults is limited (20). All co-designers completed the interview and key findings from these interviews will be presented elsewhere.

### Analysis approach

All project materials, including written materials, pictures, worksheets, and audio recordings were collated at the completion of each workshop, digitized, and stored for analysis. Audio recordings were transcribed using an online transcription service (Rev.com). Written transcripts were reviewed and utilized if they provided further data not captured by the workshop materials.

Analysis of activities occurred iteratively between workshops, with a focus on identifying insights to inform the ongoing development of the intervention. The research team involved in the delivery of workshops examined the outputs from workshop activities, transcripts, and researcher observations to identify recurring ideas and practical implications. This process drew on the principles of reflexive thematic analysis following Braun and Clarke's (35) guidance, and consistent with the principles of co-design. In line with Sanders and Stappers (19) guidance, the researchers were an active part (rather than passive observers) of the ongoing development of the intervention, and therefore analysis was both a generative and analytical process, with insights actively shaping the design of subsequent workshops and activities.

Workshop outputs were initially reviewed by (HB) and then discussed with the researchers present at the workshops (AD and AS) to ensure key ideas and perspectives were reflected in the interpretation. For example, responses to structured activities, such as activity audits or preferred intervention features, were compiled in Excel to assist in identifying recurring responses or commonly prioritized items. These summaries supported the identification of shared ideas and informed the interpretation of patterns that shaped the study. No formal inter-rater process was used, as this is not appropriate for generative co-design research. Instead, the analysis and insights gathered from previous workshops were shared with participants at the beginning of subsequent workshops to check researchers’ interpretations.

## Results

### Co-designer demographics

To prioritize the voices of the older adults in the community where the intervention would be implemented, eight ambulatory and community-dwelling older adults (mean age 74.9 ± 6.4 years, 63% female) without cognitive impairment (average T-MoCA score 20 ± 1.6 out of 22) were recruited (Table 2). One older adult co-designer opted to bring a caregiver along to the workshops for support. The remaining four co-designers were allied health professionals with 2–10 + years of experience in the profession, and a current focus of working with older adults (Table 2). See Supplementary File for workshop attendance.

**Table 2:** Co-designer demographics.

Older adult demographics and daily physical behavior									
Participant	Gender	Age	Highest qualification	Mobility aids	SEIFA score	LPA (min/day)	MVPA (min/day)	SB (min/day)	Sleep (min/day)
1	F	65	Vocational Training	Nil	3	152	33	838	417
2	F	66	Vocational Training	Nil	5	179	108	717	432
3	F	80	Vocational Training	Walking stick/frame, mobility scooter	2	108	19	653	722
4	F	74	High school	Nil	9	139	16	741	528
5	M	85	Vocational Training	Walking stick/frame	3	62	12	849	513
6	F	74	University	Nil	9	170	108	671	481
7	M	76	University	Walking stick	9	154	20	729	518
8	M	79	Vocational Training	Nil	2	115	21	815	489
Average		**75**			**5**	**135**	**42**	**752**	**513**
SD		**6**			**3**	**38**	**41**	**75**	**94**
Health professional demographics and daily physical behavior									
Participant	Gender	Age	Highest Qualification	Years in current profession	SEIFA score	LPA (min/day)	MVPA (min/day)	SB (min/day)	Sleep (min/day)
9	M	27	Bachelor of Clinical Exercise Physiology (Honours)	3–5	6	176	110	680	619
10	F	34	Graduate Diploma in Clinical Exercise Physiology	6–10	3	176	80	682	501
11	M	49	Certificate 3&4 in Fitness	+10	9	152	88	704	585
12	M	37	Master of Clinical Exercise Physiology	1–2	3	236	181	533	490
Average		**37**			**5**	**185**	**115**	**650**	**549**
SD		**8**			**3**	**36**	**46**	**79**	**63**

SEIFA, Socio-Economic Indexes for Areas; LPA, light physical activity; MVPA, moderate-vigorous physical activity; SB, sedentary behavior; SD, standard deviation; min, minutes.

#### Workshop 1

The key outputs from Workshop 1 were a series of group rules (agreement obtained in Workshop 2), and a series of mappings of a “healthy” and “unhealthy” day. The final rules were:

We will…

• Embrace open and inclusive communication.
• Cultivate a respectful and supportive environment.
• Build trust and work together.
• Engage in meaningful and focused discussions.
• Overcome communication barriers.

The 24-hour activity mapping exercise and small group discussions revealed key early insights. Co-designers recognized the importance of daily structure and purpose, though some preferred “spontaneity”, with a common view that “lack of purpose in a day” or “aimless behavior” was seen as unhealthy. Physical activity through both purposeful and incidental activities, like gardening, was valued, and many emphasized the benefit of “getting out of the house” for PA, whether through structured PA, shopping, or socializing. Two of three groups noted that “watching TV” or “quiet time” was seen as both healthy and unhealthy, depending on context (i.e., watching TV and quiet time can be relaxing as a stress reliever and prolonged participation could be seen as a negative).

Co-designers documented a typical healthy day to include around seven hours of sleep, and consistently linked “healthy food” with a “healthy” day, without prompts to consider nutrition. When sharing their sleep mapping, one older adult reflected that although their sleep was broken, it was not “unrestful” and “doesn't fuss (them) in the slightest”. This reflection offered an early insight into the older adults' resistance towards changing sleep-related behaviors.

#### Workshop 2

**Figure 2:**
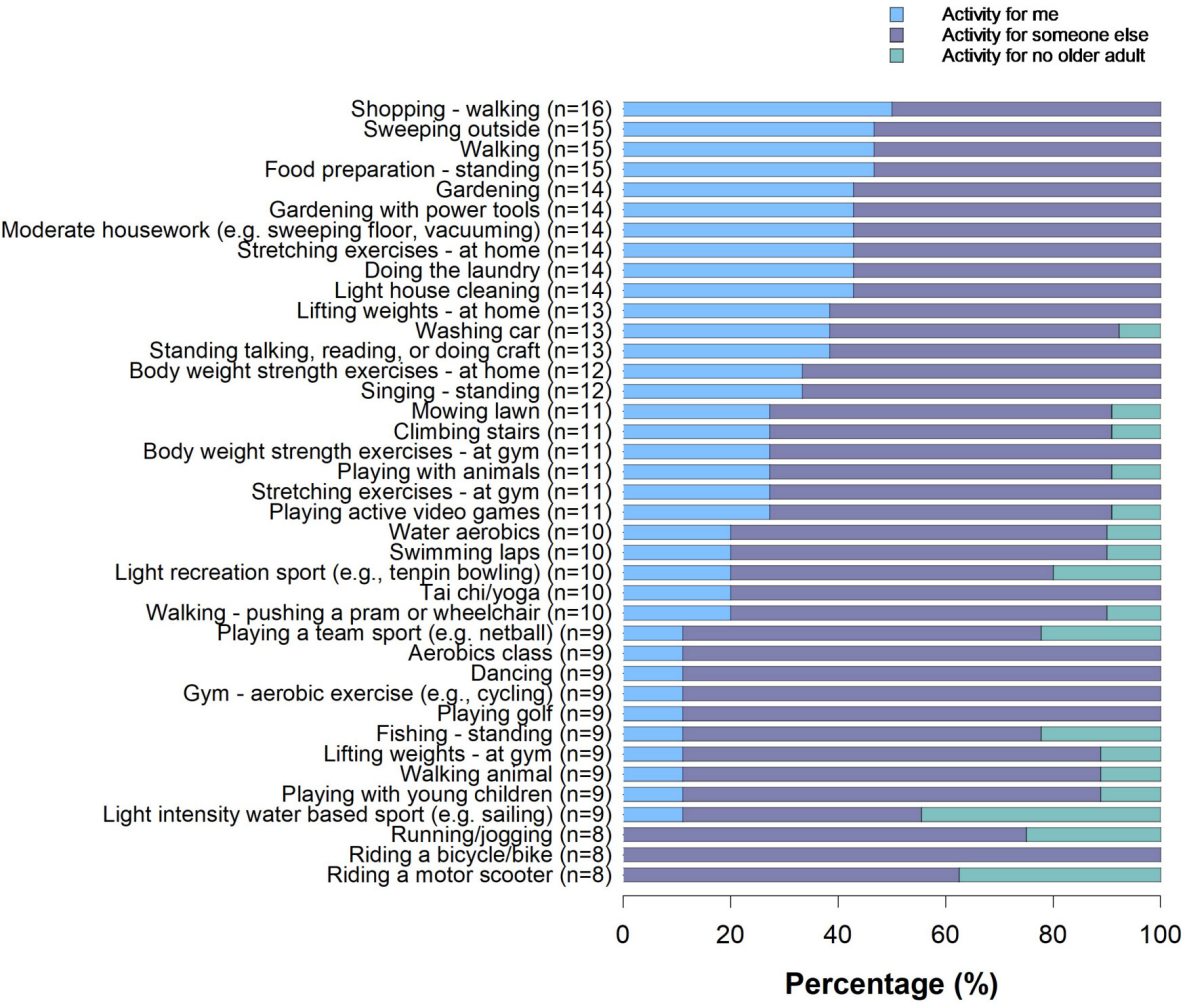
Breakdown of activities (%). Activity categories were drawn directly from the Adult Compendium of Physical Activities (30). Responses are from the older adults only. Older adults were asked to mark any activity that could be “for me”, “for someone else” or “for no older adult”, maximum responses *n* = 16. Multiple selections could be made (i.e., an older adult could select both “for me” and “for someone else”).

The key outputs for Workshop 2 included a prioritized list of physical activities that could be used as part of the PA component of the intervention (Figure 2) and insight into the behavior modification process (i.e., action planning).

During the open-ended brainstorming task, older adults found it challenging to choose behavior changes, even with suggestions and facilitator support. Many believed their days were already “very active” and “healthy”, so they could not think of small improvements. For instance, when a health professional suggested “you could take some breaks when reading and stretch”, an older adult resisted, replying “but, I’m comfy and warm lying in bed doing this”.

In a group discussion on the “Job Story” task, the third, most detailed option, which, in addition to the SB and PA, included “where”, “with”, “for how long”, “as long as I have”, and an open-ended prompt of “and”, was the generally preferred option. The added level of complexity was valued: for instance, one older adult reported that while they may want to go to the shops more to walk, they need a support worker to attend and to use their walker, which needs prior consideration. The additional detail of “as long as I have” they felt, would prompt this consideration. Although, it was noted that too much detail could be a barrier to completing the task, and therefore the latter detail was to be an optional field in the intervention. The need for activity personalization and genuine consideration for the context of a behavior was further highlighted in discussions about daily activities, like “watching TV”. An older adult emphasized the type of TV matters, seeking to differentiate between the “trashy TV” that others watch, and the more “engaging TV” that they watch, suggesting that the relevance of certain activities in behavior change are not consistent and needs consideration.

**Figure 3:**
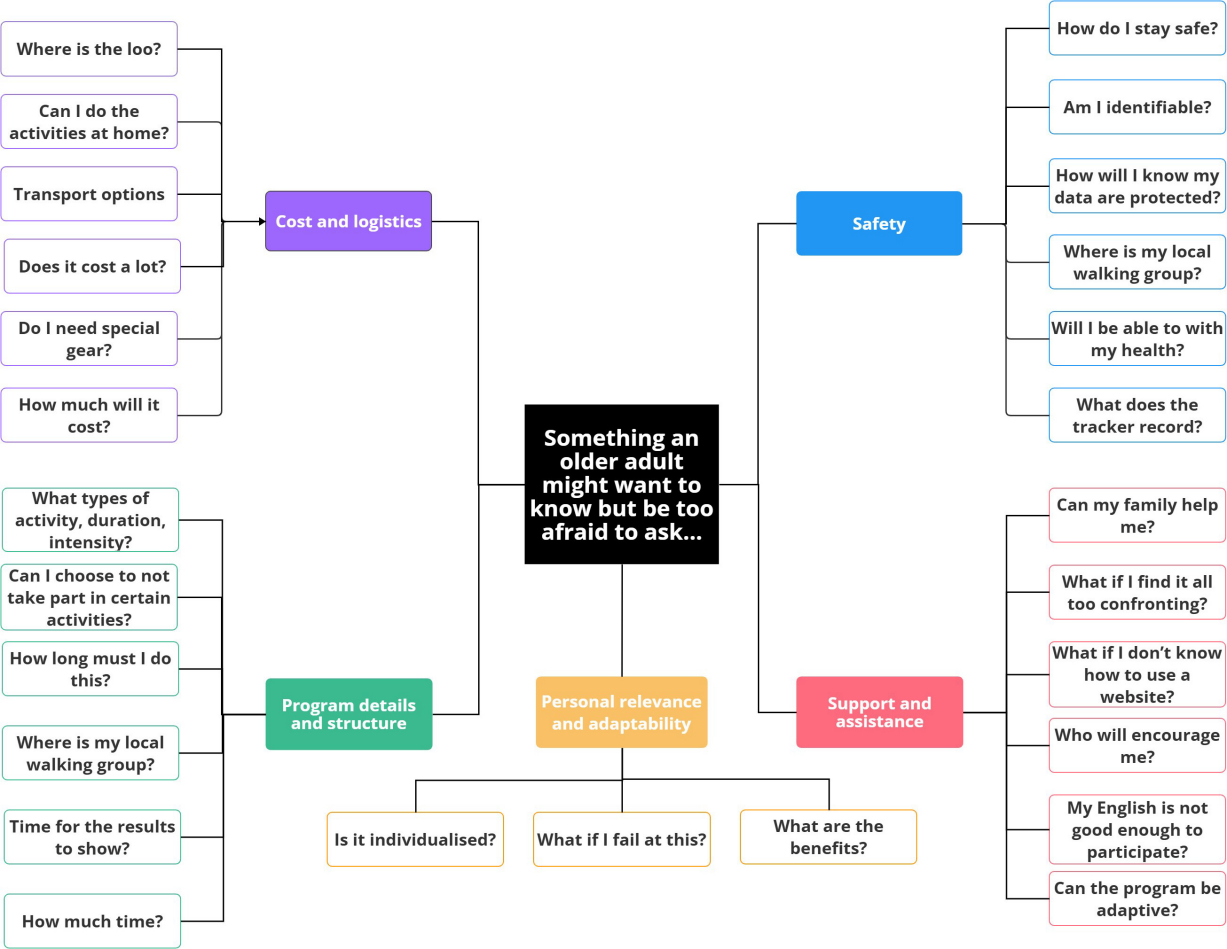
Thematic breakdown of questions on cost and logistics, program details and structure, personal relevance and adaptability, safety and support and assistance.

The final activity asked co-designers to develop questions based on the prompt: “Something an older person might want to know but be too afraid to ask when participating in this program is…”. This activity generated primarily logistical questions covering key topics, such as: (1) personal relevance and adaptability; (2) cost; (3) logistics and safety; (4) program details and structure; and (5) support and assistance (Figure 3). Questions like “what if I find it too much and want to quit?”, and “What if I fail at this” were raised and discussed between co-designers. During this, and future discussions, the ability to adjust the pace of the intervention appeared important.

#### Workshop 3

The primary workshop outputs built on the co-designers' preferences for the level of detail they wanted in the intervention. Key insights included a preference for gradual behavior modifications over time, a desire for both the provision of behavior change suggestions and personal autonomy, and the resistance of the older adults towards sleep focused behavior changes.

A key discussion point identified from the paper-based action planning task related to co-designer's feelings about whether to implement a single behavior change or multiple changes simultaneously, with a tendency towards gradual progression. This tendency suggested a need for weekly progressive action planning as part of the intervention, however, when taken together with co-designers worry about “failing” identified in Workshop 2, the capability to skip weeks as needed was important. One community participant observed benefits and challenges in massed vs. staggered behavior change:

“If people sat down and looked at an overall of the program and go, it’s 12 weeks and they go, oh my God, I could be dead by then. Or, oh there’s four things to change. Oh look, that’s a lot of effort, a lot of work. Maybe it needs to be broken into steps. So maybe the first three weeks you change one thing, the next three so that you've got little ticks of achievements that motivates you to keep going to the next one. If you just give them the whole big picture to start with, it may be a bit daunting and they may go that’s just way too much, I can't do all those, it’s too much. Whereas if you gave them something for a couple of weeks, they'd probably manage that and then they can build from there as their confidence grows as well.”

There was general agreement on the importance of providing specific PA examples for future participants to choose from, along with the option for customization. Further, the paper prototype exercise underscored the need for context-specific filtering of intervention elements as co-designers, particularly the older adults, struggled when presented with inappropriate behavior matches, for instance the nonsensical possibility of selecting “go for a jog” as a pair with “while I am watching TV I will …”.

In contrast to the PA prototype, older adults struggled to engage with the role-play aspect of the sleep prototype and instead defaulted to relating sleep to their personal experiences. Facilitators were surprised by the strong resistance to sleep changes. Two older adults (from separate tables) reported that they did not believe there to be enough information about sleep to improve it or that there is “no cure” for poor sleep. Similarly, another table concluded that sleep is much more complex and personal than PA.

To finish, the approach to understanding what makes information comprehensible largely aligned with established principles of information design. Key elements included using readable font sizes and styles, incorporating pictures and diagrams, ensuring sufficient colour contrast for both diagrams and text, and avoiding **s**cientific language or overly lengthy explanations.

#### Workshop 4

The primary outcomes of Workshop 4 orientated around co-designers' experience and evaluation of the initial paper-based website.

From the paper-based website activity, select health professionals tended to provide feedback on layout and the extent of information being asked of future users, while older adults focused on elements within their perceived realm of familiarity and expertise, such as font size, clarity of language, and whether the representation of imagery aligned with their demographic (e.g., imagery that accurately reflects older adults). When asked about desired website features to be presented following an action plan, co-designers emphasized the need for elements such as positive feedback and reinforcement, a summary of recently completed steps, progress tracking, visibility of goals and progress, and access to further education and support. In response, the research team incorporated elements like a summary page before confirming each weekly action plan, along with a progress bar and a comprehensive summary of all action plans on the homepage.

Commonalities among co-designers' preferences for website and intervention features, identified by frequency, were categorized as: “education”; “an ability to contact the researcher”; “providing a summary of weekly behavior selections”; “displaying intervention progress”; and “providing links to relevant organizations and services”. The research team integrated these prioritized features into the first digital prototype, which was subsequently explored in Workshop 5.

#### Workshop 5

This workshop provided co-designers their first opportunity to view and give feedback on the digital prototype of the intervention website. Co-designers expressed preferences for intuitive navigation, clear and specific instructions, reputable educational resources, and demonstrated that the prototype could be learned and navigated with practice.

Despite the use of role-play, resistance to sleep strategies resurfaced, with some older adult co-designers discussing that improvements from sleep due to PA would suffice. However, they acknowledged the importance of including sleep strategies for a broader user base, highlighting that while not deemed personally important, there was appropriate consideration of others.

The visual layout of the digital prototype was positively received, though co-designers identified a need for clearer step-by-step instructions. Confusion arose when navigating drop-down menus and the need for context-specific filtering to minimize illogical PA options for certain SBs (e.g., “while watching TV… ‘I will go for a walk’”) resurfaced from Workshop 2. Filtering logic to minimize inappropriate options was implemented for Workshop 6. Additional prompts for identifying SBs, highlighted as important during the paper-based prototype in Workshop 4, were included in this version for future targeting. However, this feature when used in the digital prototype caused consistent confusion and was removed prior to Workshop 6.

The role-play instructions gradually required more autonomy throughout the workshop, and it was apparent that co-designers were beginning to “learn” how to navigate the website. This was evident as older adult co-designers started instructing other group members on how to complete certain steps or access certain website features. When prompted by the group's health professional about their ability to use the website on a weekly basis, one pair of older adults shared that, “most people would be generally okay, but it may take some practice to get used to it”.

Following recommendations from Workshop 4, educational resources were integrated into the prototype, featuring concise summaries (∼80 words) with links to more comprehensive evidence-based materials. Co-designers responded positively, with one health professional noting, “I really like the resources and the provision of the shorter and longer option,” reflecting a common sentiment that flexibility caters to different user preferences. Navigation was generally intuitive, as evidenced by comments like “that's easy” while exploring tabs; though some confusion arose when trying to return from external resources, underscoring the need for health professional support and regular check-ins during the intervention. To promote resource engagement and relevancy, the website was designed to present users with an educational resource that aligned with the weekly action plan focus (e.g., walking or sleep hygiene tips), which was well-received, with one older adult remarking, “oh that's good, isn’t it,” when noticing the relevant content. Co-designers strongly preferred “scientifically backed” information over influencer or blog content, and prioritizing credibility over format.

#### Workshop 6

Between Workshop 5 and 6, further iterative refinements were made to the website and a near final prototype was presented to the co-designers. Co-designers responded positively to the iterative changes to the website, and it was clear from responses that older adults were finding the website and the check-in process easy to understand and the design palatable, with comments such as “…ah ha, remembered that from last time”, “…I like the look of that” (referring to the dashboard), and “…Oh, ooo, look, look, look, look! This is fabulous please sign me up!” (referring to a purpose-built educational resource of a home-based strength exercise routine to be provided if strength training at home was selected for the respective action plan).

During the light lunch and socialisation, conversation was positive, with one older adult sharing the following reflection:

“I’ll tell you one thing that I did get out of it. Besides having fun coming to this. Is (sic) that I did take home and try the regular sleep pattern, get up time. It does help.”

## Discussion

This study forms the first part of the larger program of work developing, implementing, and evaluating a novel web-based 24-hour intervention called Small Steps, and outlines the “how to” of the co-design process, including the activities involved, the pivotal outcomes identified, and reflection on their impacts to the project.

### Co-design captured unique perspectives

Significant iterative improvements were made to the intervention based on the unique perspectives and needs of the older adults. It became clear that co-designers valued both support and autonomy for the action planning element of the intervention (36). Further, a need for detailed selections was preferred. These findings align with the extant action planning literature, emphasizing the importance of balancing guidance and personal choice (37, 38). Moreover, a step-by-step approach was preferred, allowing for gradual behavior changes across the duration of the intervention to avoid feelings of becoming overwhelmed. These are consistent with previous PA approaches for older adults (39). Action planning detail and frequency preferences were integrated into the final intervention, although to address concerns around “failure”, skipping weeks was made possible, despite this potentially compromising the research data that could be collected. To further support personalisation, the software placed personally preferred activities at the top of the action planning list.

The iterative design process for the intervention also highlighted the importance of incorporating individual choice and personalization, as revealed through workshop outputs. In Workshop 1, co-designers expressed a general awareness of the need for daily structure and purpose, though preferences varied, with some co-designers' favoring spontaneity over rigid scheduling. This finding underscored the necessity for the intervention to allow flexibility in behavior change, accommodating both structured and more spontaneous approaches to daily activities. In Workshop 2, when co-designers were prompted to think about replacing a current activity with a more active one, many struggled to generate ideas, even with facilitator support. This uncovered the need for the intervention to offer tailored suggestions for behavior change opportunities while maintaining autonomy for users to choose activities that suited their personal preferences. In addition to tailored suggestions, the functionality to input custom behaviors was afforded to ensure true personalization and autonomy.

Resistance to changes in sleep patterns became evident during Workshop 3, further influencing the design of the end-product in ways that the authors had not expected. Co-designers expressed hesitation and skepticism regarding the modification of sleep behaviors, citing a belief that there is limited information available on how to improve sleep or that there is “no cure” for sleep difficulties. Many viewed sleep as a more complex, deeply personal, and self-manageable issue than PA, with some co-designers finding it challenging to engage in role-playing scenarios that involved altering their sleep habits. Similar barriers to seeking treatment for sleep disorders have been reported in populations from the United Kingdom (40, 41). This resistance to making changes to sleep prompted the researchers to make sleep-related interventions optional within the final intervention product, allowing future participants the freedom to focus their action planning on either PA, sleep, or a combination of the two behaviors, depending on their preferences. To address concerns about the lack of evidence-based guidance on sleep, the use of sleep hygiene scores and data visualization tools during a baseline intervention session with a health professional were to be utilized. This would ensure that any discussions about changing sleep-related behaviors were grounded in objective and personalised data, thereby enhancing future participants' confidence in the intervention's ability to support meaningful change. Interestingly, a gradual shift in the co-designers' openness to modify sleep patterns underscored the effectiveness of the participatory co-design in developing this intervention.

### Innovative co-design techniques promoted co-designer insights

To stimulate creative design thinking, a variety of innovative methods were employed. Initially, website development began with a paper-based mock-up of the intervention, allowing co-designers to explore the behavior changes process (i.e., action planning). In subsequent workshops this evolved into co-designers interacting with digital prototypes, using screen- and voice recordings to capture the co-designers' real-time experiences. The richness of data gathered using these methods may not have been possible through verbal feedback alone, and reflects similar findings on the importance of undertaking creative making activities and prototyping (42, 43). Progression from paper to digital prototyping exposed discrepancies in co-designer opinions from undertaking a paper-based task and translating that to a digital interface. For example, during paper-based prototyping, co-designers suggested it would be beneficial to identify new SBs each week that could be targeted in future weeks, but their subsequent experience with the digital prototype indicated this feature was repetitive and confusing, leading to its removal in the final product.

To encourage design-based thinking, game-based activities such as role-play and third-person scenarios were used (44). These techniques allowed co-designers to embody a different perspective (other than their own) and step outside their own experiences to consider how others might interact with the intervention. This approach not only created a sense of psychological safety by reducing the focus on self, but also facilitated the expression of tacit knowledge through creative activities (19). The use of iPad screen and audio recordings further reduced the burden of co-designers and facilitators to scribe or document responses and allowed experiences to be captured passively. Together, these strategies allowed for deep insights into the user experience and iterative refinement of the end product.

### Project ownership and engagement

A key feature of the co-design process was a fostering of ownership among co-designers for the process and product being developed (25). For example, co-designers drafted and agreed on rules of engagement, which were displayed on each table for the duration of the project. Researchers also upskilled co-designers on relevant content to ensure feedback was evidence-based. Co-designers, as well as the research team, participated in collaborative activities, and their impact on the wider project was continually displayed back to them. There were many examples across the duration of the workshops that indicated participation in the workshops shifted from a transactional relationship (of being involved in a study) to an ongoing relationship. For example, there was a 100% retention rate across the workshops among older adults, aligning with previous successes (45). Co-designers also autonomously contributed to the intervention development between sessions. Specifically, a health professional often contacted the lead facilitators to provide further clinical insights into the intervention being developed. Further, one older adult presented the lead facilitator with their ideas for a community center that could foster inclusion and activity for older adults, another provided two short writings about personal health experiences, and another wrote a poem and song about their experience with the workshops, detailing the positives and challenges of developing an intervention appropriate for all.

“…I loved the workshop people all, and hope we keep the friendship rule. To greet each other when we meet with juicy gossip, what a treat.”

### Limitations

Key strengths of the co-design approach included descriptively following the Double Diamond model, which allowed for a shift between divergent and convergent thinking. Similarly, the inclusion of all stakeholder groups together in a workshop series at the same time and in the same place, appeared to foster a sense of value and project ownership. However, there are several limitations that should be considered. For example, it is possible that the inclusion of all end-users together in a single workshop may have led to power imbalances between older adults, health professionals, IT developers, and researchers. Efforts were made to ensure the older adult voice was the predominant one, by recruiting older adults to health professionals in a ratio of 2:1. This research focused on engaging older adults and healthcare professionals to identify key insights that could shape a scalable and testable product. In addition, sampling bias may have been introduced by convenience sampling older adults. This may have resulted in unintentionally recruiting participants who were more socially engaged and with higher digital literacy levels. As a result, the Small Steps intervention may not be generalizable for the larger population. Thirdly, while carers and partners were welcome to participate in the co-design process, their involvement was not the focus of this phase. Future work should more explicitly include caregivers to ensure the intervention meets the needs of older adults requiring additional supports. Finally, while the design principles developed in this study align with much of behavior change literature, the Small Steps intervention has not yet been trialled. Further research is required to determine the Small Steps effectiveness first through local intervention and implementation before further adaptation and scaling.

## Conclusions

This study outlines the co-design process undertaken to create an individualized 24-hour time-use intervention called Small Steps. Engaging a single diverse group of older adults and allied health clinicians, as well as researchers and software developers in a participatory design process, we iteratively developed the Small Steps intervention. The co-design approach captured co-designers' unique perspectives, leading to iterative improvements focused on meeting the specific needs of its intended users. Key factors contributing to the co-design included flexibility in the design process, fostering a supportive environment, and empowering co-designers through activities that guided and stimulated their thinking. These elements not only helped to shape the development of Small Steps, but reinforced the value of co-design in developing personalised interventions for older adults.

## Data Availability

The datasets presented in this article are not readily available because ethics approvals preclude sharing of identifiable data. Requests to access the datasets should be directed to ashleigh.smith@unisa.edu.au.
