# Supplementary material for: Co-design of a digital 24-hour time-use intervention with older adults and allied health professionals
Source: Front Digit Health. 2025 May 23;7:1544489. doi: 10.3389/fdgth.2025.1544489 (PMC12141298; doi:10.3389/fdgth.2025.1544489)
Supplement: Supplementary file 1 [file Datasheet1.docx]

**Title:** Co-design of a digital 24-hour time-use intervention with older adults and allied health professionals – Supplementary files.

**Authors:**

Henry T Blake^a^, Aaron Davis^b^, Maddison L Mellow^a^, Melissa Hull^a^, Bethany Robins^a^, Kate Laver^c, d^, Dorothea Dumuid^a^, Timothy Olds^a^, Hannah AD Keage^e^, Lui Di Venuto^f^, Ashleigh E Smith^a^*

**Affiliations:**

1. Alliance for Research in Exercise, Nutrition and Activity (ARENA) Research Centre, Allied Health and Human Performance, University of South Australia, Adelaide, SA, Australia
2. Australian Research Centre for Interactive and Virtual Environments (IVE), UniSA Creative, University of South Australia, Adelaide, SA, Australia
3. Division of Rehabilitation, Aged and Palliative Care Service, Southern Adelaide Local Health Network, Bedford Park, SA, Australia
4. Caring Futures Institute, College of Nursing and Health Sciences, Flinders University, Bedford Park, SA, Australia
5. Justice and Society, University of South Australia, Adelaide, SA, Australia.
6. Onkaparinga City Council, Adelaide, SA, Australia

***Correspondence:** Ashleigh E. Smith, Alliance for Research in Exercise, Nutrition and Activity (ARENA) Research Centre, Allied Health and Human Performance, University of South Australia, GPO Box 2471, Adelaide, South Australia 5001. Email: [Ashleigh.smith@unisa.edu.au](mailto:Ashleigh.smith@unisa.edu.au)

**Supplementary Material:**

Supplementary Table 1. Workshop attendance

|  |  | Workshop 1 | Workshop 2 | Workshop 3 | Workshop 4 | Workshop 5 | Workshop 6 | Post workshop evaluation | **Percent attendance** |
| --- | --- | --- | --- | --- | --- | --- | --- | --- | --- |
| **Participants** | Community expert 1 | • | • | • | • | • | • | • | **100** |
|  | Community expert 2 | • | • | • | • | • | • | • | **100** |
|  | Community expert 3 | • | • | • | • | • | • | • | **100** |
|  | Community expert 4 | • | • | • | • | • | • | • | **100** |
|  | Community expert 5 | • | • | • | • | • | • | • | **100** |
|  | Community expert 6 | • | • | • | • | • | • | • | **100** |
|  | Community expert 7 | • | • | • | • | • | • | • | **100** |
|  | Community expert 8 | • | • | • | • | • | • | • | **100** |
|  | Exercise physiologist 1 | • | • | • | • | • | • | X | **86** |
|  | Exercise physiologist 2 | • | • | • | • | • | • | • | **100** |
|  | Fitness Trainer 1 | • | • | X | X | • | • | • | **72** |
|  | Exercise physiologist 3 | • | • | • | • | • | • | • | **100** |
|  | Researcher 1 | • | • | • | • | • | • |  | **100** |
|  | Researcher 2 | • | • | X | X | X | X |  | **33** |
|  | Researcher 3 | • | • | • | • | X | • |  | **83** |
|  | Researcher 4 | X | X | X | X | • | • |  | **33** |
|  | Researcher 5 | X | X | • | • | • | • |  | **66** |

*Note. • Participant present at the workshop, X = participant absent at the workshop. Research staff rotated across the workshops, with only HB (lead facilitator) present at all workshops.*

**Supplementary Table 2.** Workshop Run Sheets.

| Small Steps Workshop 1 | | | |
| --- | --- | --- | --- |
| Activity | **Facilitator** | **Participation** | **Materials** |
| Welcome | HB |  |  |
| Background – Upskill What is Small Steps | HB |  | PowerPoint |
| Co-design introduction | AD |  |  |
| Icebreaker – name tag creation and special skill | AD | Whole group | Name tags, pens |
| A day in the life | HB | Individual | A day in the life template |
| Healthy and unhealthy days | HB | Individual | Healthy and unhealthy day templates |
| Sharing and collating healthy and unhealthy days at table | HB | Table groups | Scale balance template |
| Whole group share with reflection | HB/KL | Whole group | Butchers paper, markers |
| Great and terrible collaborations | HB | Table groups | Great and terrible template |
| Share and collect set of “rules”  Gradient of agreement and acceptance | HB | Whole group | Completed great and terrible templates |
| Close | HB |  |  |

| Small Steps Workshop 2 | | | |
| --- | --- | --- | --- |
| Activity | **Facilitator** | **Participation** | **Materials** |
| Welcome | HB |  |  |
| Recap (inc. rules consensus) and upskilling | HB/AD |  | PowerPoint |
| Activity audit | HB | Individual | Activity audit template |
| Activity replacement brainstorm | HB | Individual | Activity replacement template |
| Job story activity | HB | Individual | Job story template |
| Reflection on job story activity | AD | Table groups | Butchers paper, markers |
| Questions if enrolled in the intervention | HB | Individual | Question card template |
| Close | HB |  |  |

| Small Steps Workshop 3 | | | |
| --- | --- | --- | --- |
| Activity | **Facilitator** | **Participation** | **Materials** |
| Welcome | HB |  |  |
| Role play website paper prototype (PA) | Health professionals | Table groups | Scenario, paper prototype |
| Role play website paper prototype (sleep) | Health professionals | Table groups | Scenario, paper prototype |
| Action planning process discussion | Health professionals | Table groups | Butchers paper, markers |
| Simple and complex information comparison | HB | Individual | Simple and complex template |
| Discuss above task | Researchers | Table groups | Completed simple and complex template |
| Close | HB |  |  |

| Small Steps Workshop 4 | | | |
| --- | --- | --- | --- |
| Activity | **Facilitator** | **Participation** | **Materials** |
| Welcome | HB |  |  |
| Website booklet | HB | Individual | Website booklet |
| Design website dashboard | HB | Individual | Design sheet |
| Close | HB |  |  |

| Small Steps workshop 5 | | | |
| --- | --- | --- | --- |
| Activity | **Facilitator** | **Participation** | **Materials** |
| Welcome | HB |  |  |
| Exploring new website – role play 1 | Researchers and Health Professionals | Partners | Exploring sheet (Act. 1) |
| Teaching new website – role play 2 | Researchers and Health Professionals | Partners | Teaching sheet (Act. 2) |
| Independent role play – role play 3 | Researchers and Health Professionals | Individual/ Partners | Independent role play sheet (Act. 3) |
| Close | HB |  |  |

| Small Steps Workshop 6 | | | |
| --- | --- | --- | --- |
| Activity | Facilitator | Participation | Materials |
| Welcome | HB |  |  |
| Overview of previous co-design | HB |  | PowerPoint |
| Prototype testing (including resource review) | Researchers and Health Professionals | Table groups | iPads, scenario, task sheet |
| Thanking for co-design   - Any final comments? | HB |  |  |
| Close | HB |  |  |


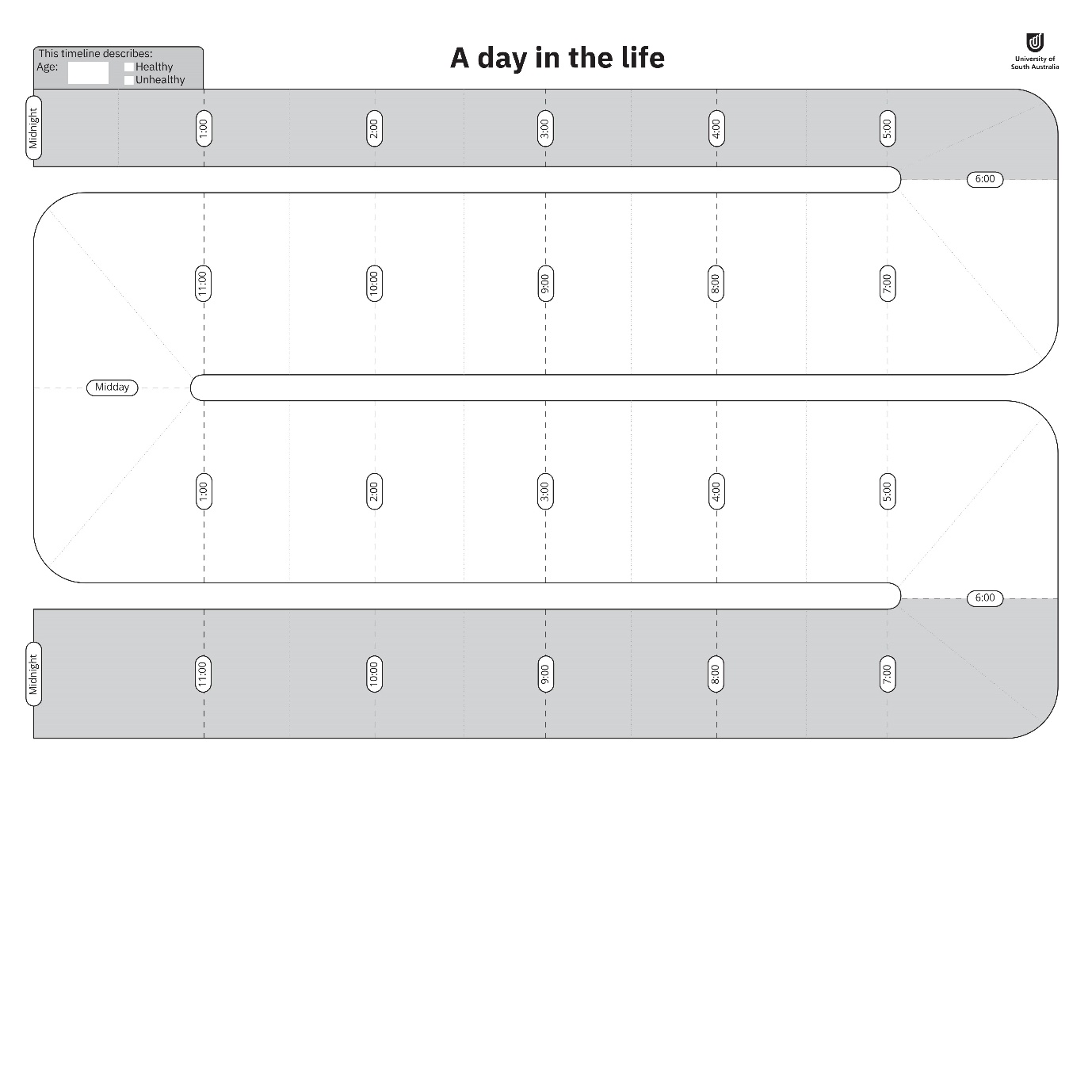


**Supplementary Figure 1**. Workshop 1. A day in the life worksheet. Workshop participants illustrated their own understanding of what constituted a ‘healthy day’ and an ‘unhealthy day’ by writing a breakdown of activities in each of the 1-hour segments. Overall results were compiled and discussed at each table.


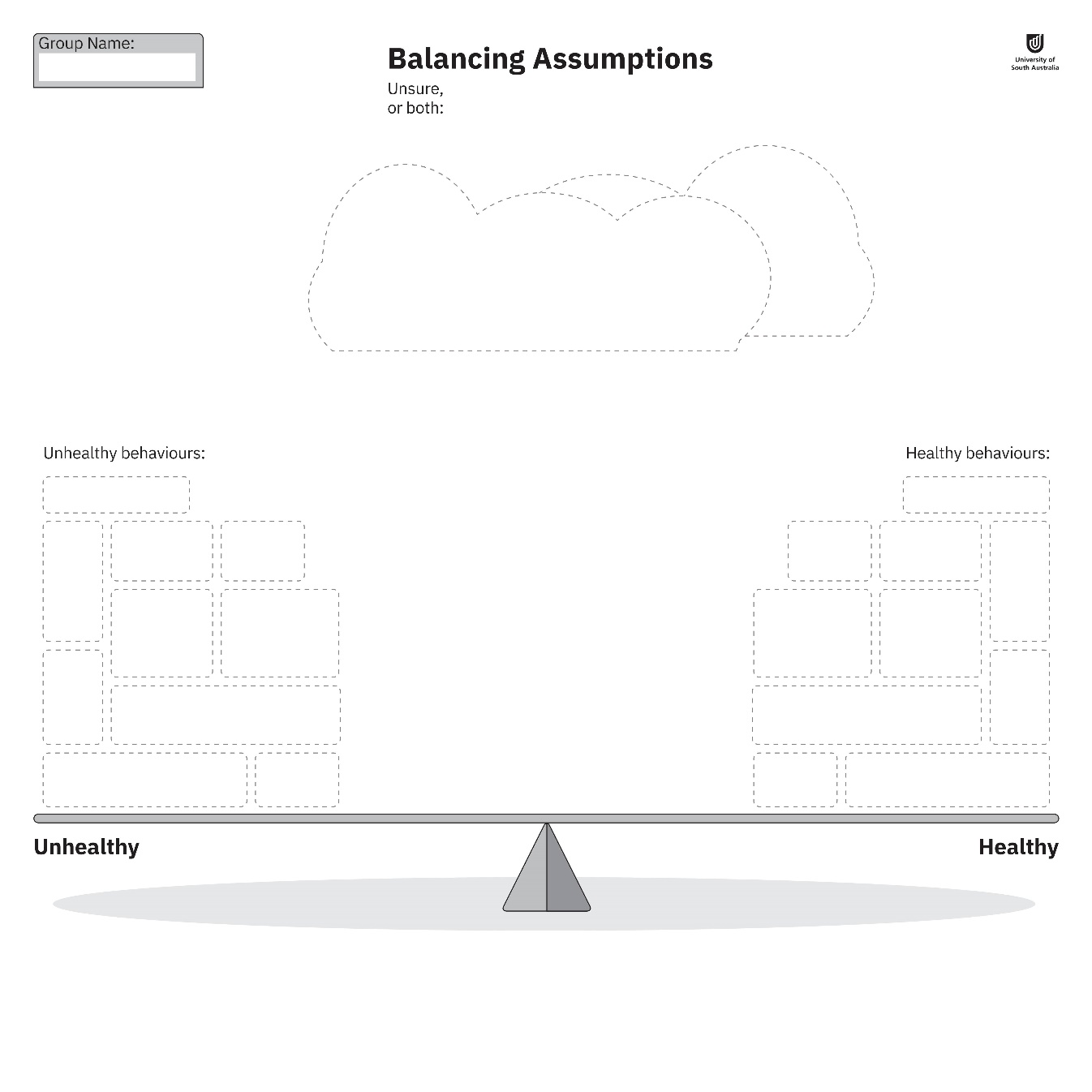


**Supplementary Figure 2.** Workshop 1. Balancing assumptions worksheet. At tables, within small groups, participants listed behaviors they believe to be healthy and unhealthy, as well as those they we unsure of or thought could be considered both healthy and unhealthy. Each table verbally reported their decisions to the wider group for sharing and further discussion.


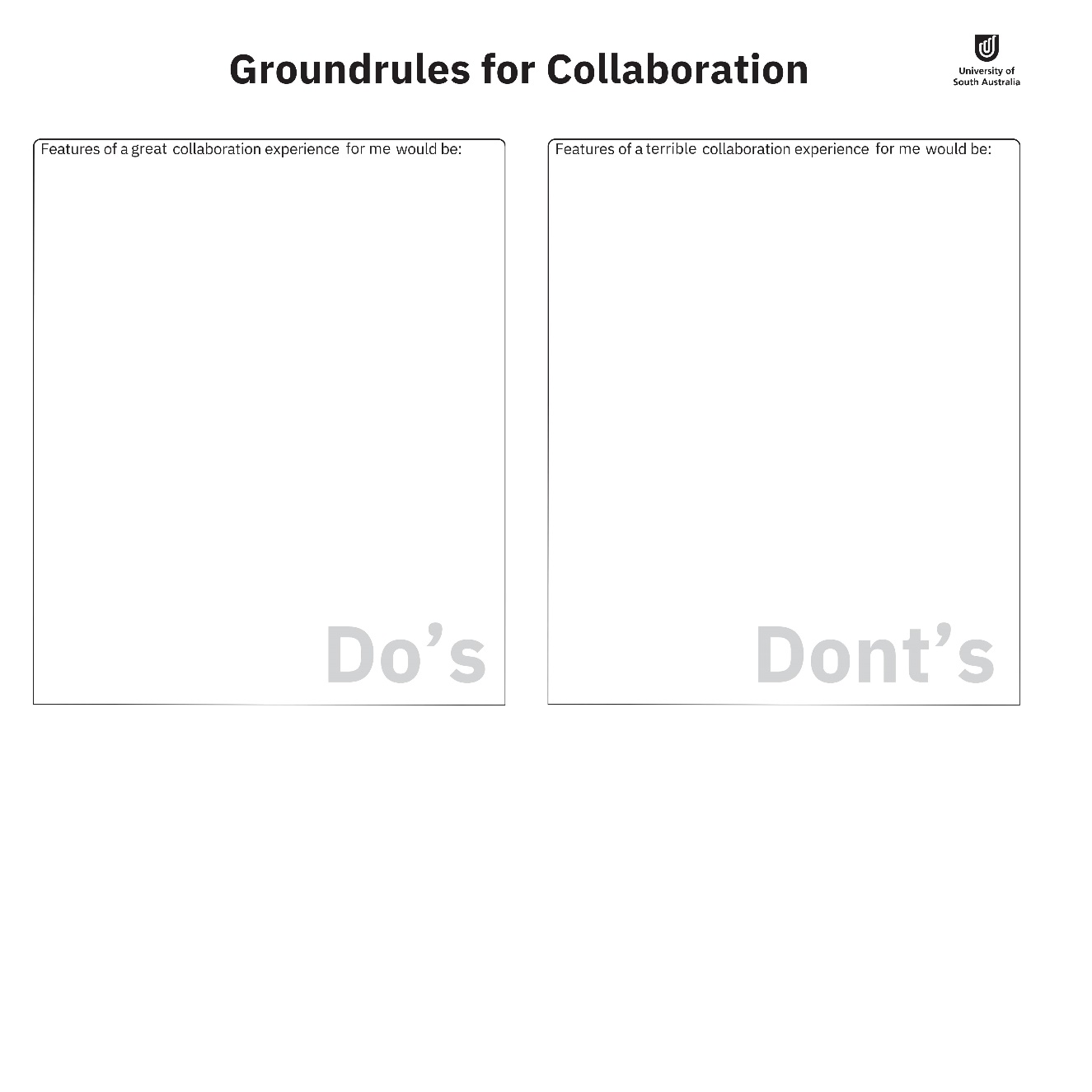


**Supplementary Figure 3.** Workshop 1. Individual ground rules for collaboration worksheet. Participants individually and then in groups listed all the features of a good collaboration experience and a negative collaboration experience.


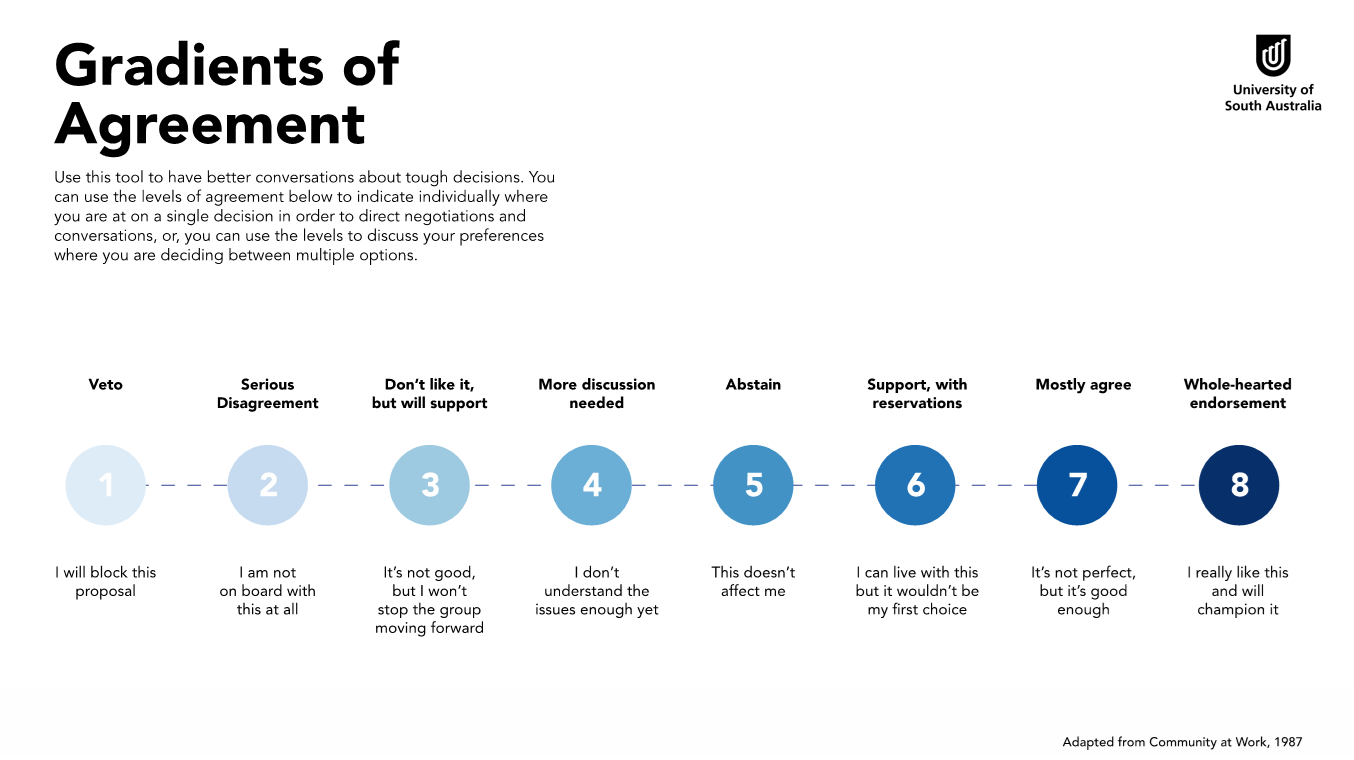
**Supplementary Figure 4.** Workshop 2. Gradients of Agreement scale used to capture participants' agreeance (or lack thereof) towards the five workshop rules. At tables, participants individually reported their level of agreement and then tables verbally reported these to the wider group. A level of 6 or above was determined to be sufficient to implement the group rule, which was achieved. This scale was adapted from Community at Work (Kaner, S. (2014). *Facilitator's guide to participatory decision-making*. John Wiley & Sons, Incorporated).

S**upplementary Figure 5.** Workshop 2. Activity audit required participants to select whether a proposed physical activity is suitable for themselves (as an older adult), for another older adult, or not suitable for an older adult. While also completed by Health Professionals, who were told to answer based on whether they think they are appropriate for older adults, only results from the older adults were interpreted and presented. Phyical activity options identified through the Physical Activity Compendium (Ainsworth, B. E., Haskell, W. L., Herrmann, S. D., Meckes, N., Bassett, D. R., Jr., Tudor-Locke, C., Greer, J. L., Vezina, J., Whitt-Glover, M. C., & Leon, A. S. (2011). 2011 Compendium of Physical Activities: a second update of codes and MET values. Med Sci Sports Exerc, 43(8), 1575-1581. <https://doi.org/10.1249/MSS.0b013e31821ece12>).


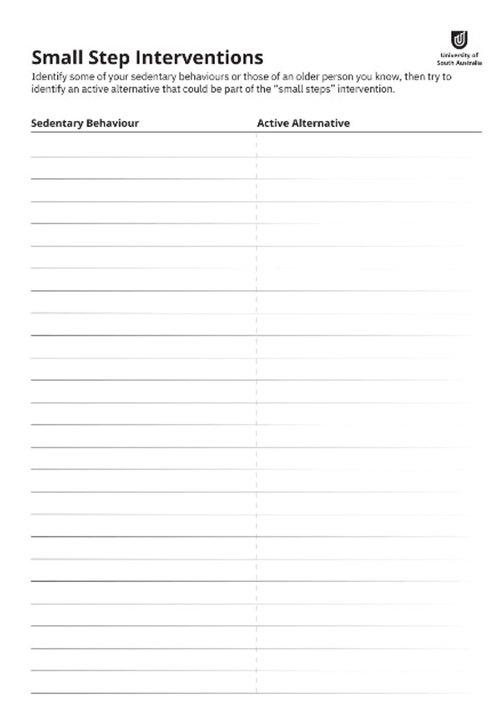


**Supplementary Figure 6.** Workshop 2. Worksheet for open-ended brainstorming of behavior alternatives. Participants individually, although able to discuss their thoughts/progress with tables members, identified sedentary behaviors that an older person may engage in and then physical activity replacements that might make up part of the Small Steps intervention to replace them.

**Supplementary Figure 7.** Workshop 2. Job Story template worksheet. Participants identified a single sedentary behaviour (i.e., When I am) and completed each of the three response boxes, each varying in the level of detail required. Progressing through the level of detail required was intended to aid participants in experiencing and identifying the desired level of detail that would be preferred when deciding on behavior changes as part of the intervention.


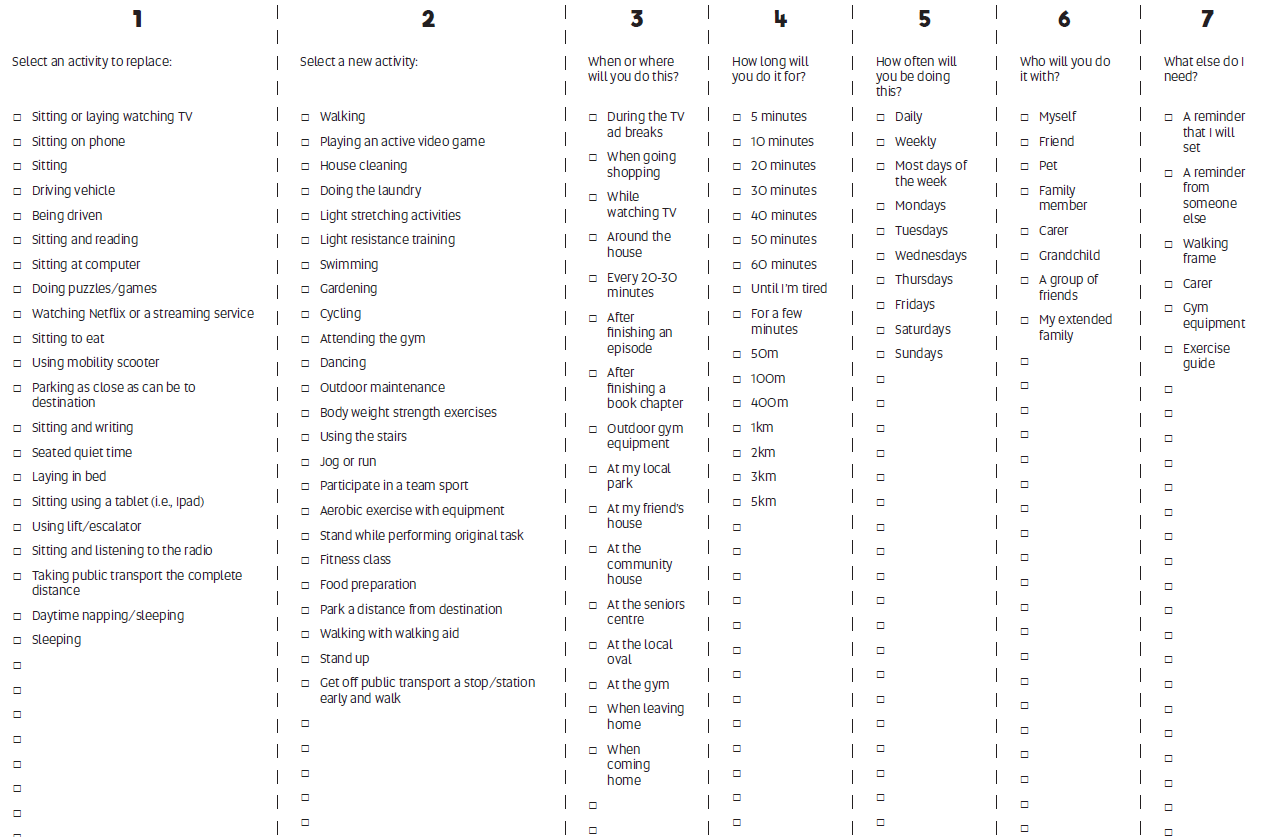

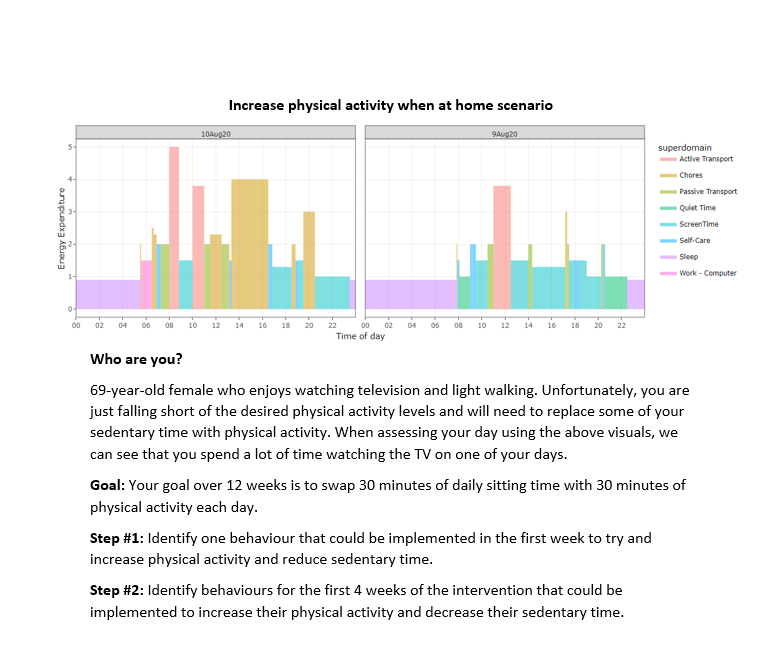


**Supplementary Figure 8.** Workshop 3. Example role-play scenario (top image) and associated paper-based action planning (bottom image) for physical activity. Groups (two older adults and one health professional) were guided to complete the paper-based action planning based on the provided scenario. The figure provided in the scenario was a mock visualization of the physical behaviour of the individual described in the scenario.

**
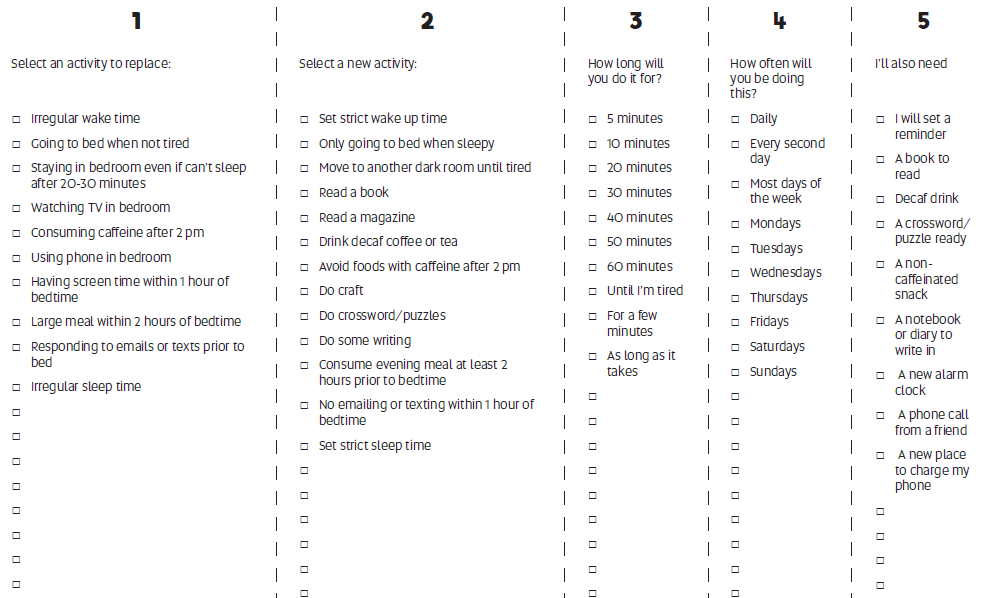

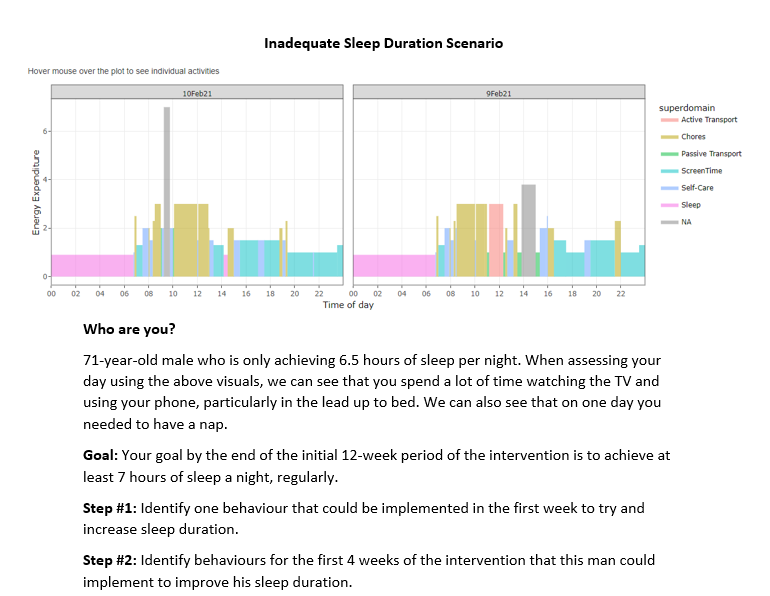
**

**Supplementary Figure 9.** Workshop 3. Example role-play scenario (top image) and associated paper-based action planning (bottom image) for sleep behaviour. Groups (two older adults and one health professional) were guided to complete the paper-based action planning based on the provided scenario. The figure provided in the scenario was a mock visualization of the physical behaviour of the individual described in the scenario.


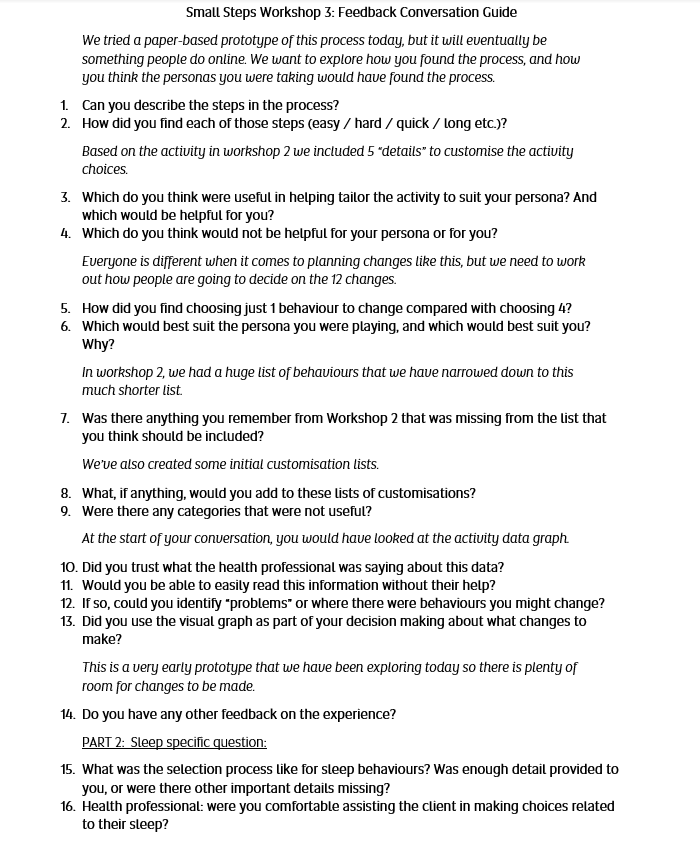
**Supplementary Figure 10.** Workshop 3. Semi-structured questions that could be used by health professionals and/or workshop facilitators that ‘could’ be used after completing the paper-based action planning to get further insight from participants.

**Supplementary Figure 11.** Workshop 3. Worksheets to prompt co-designers to consider, individually (top) and then as a table (bottom), examples of complex and hard to understand and simple and easy to understand information
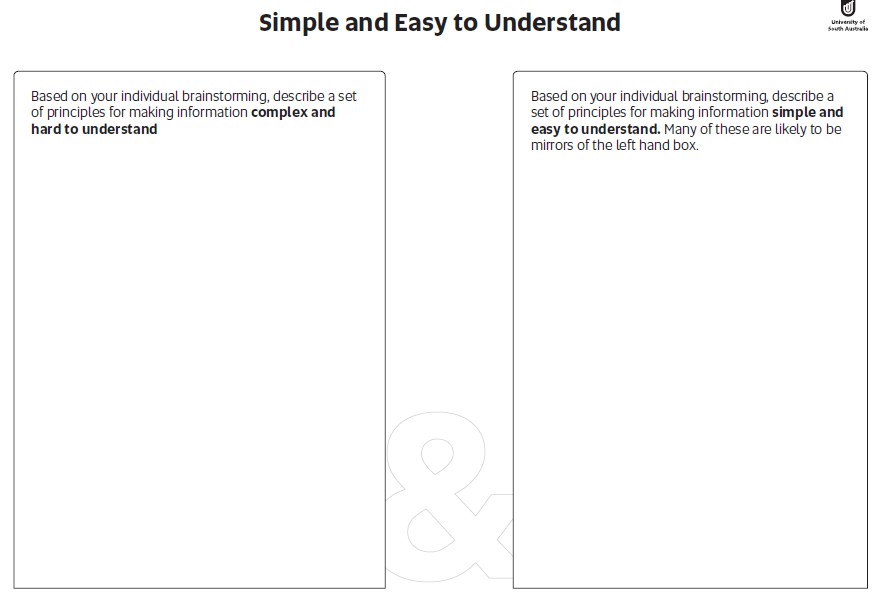

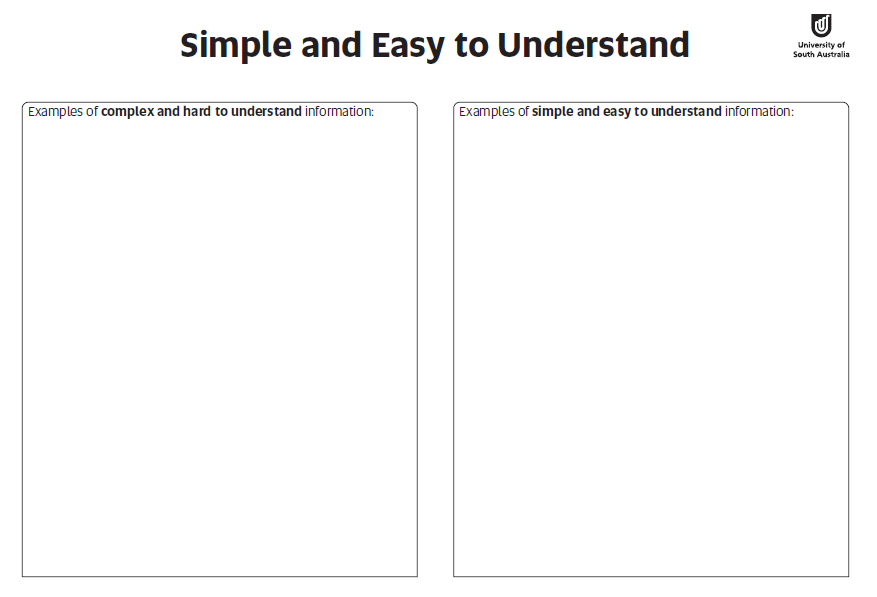
. Insight from this were intended to provide initial guidance regarding the ‘look’ and ‘feel’ of the intervention website.


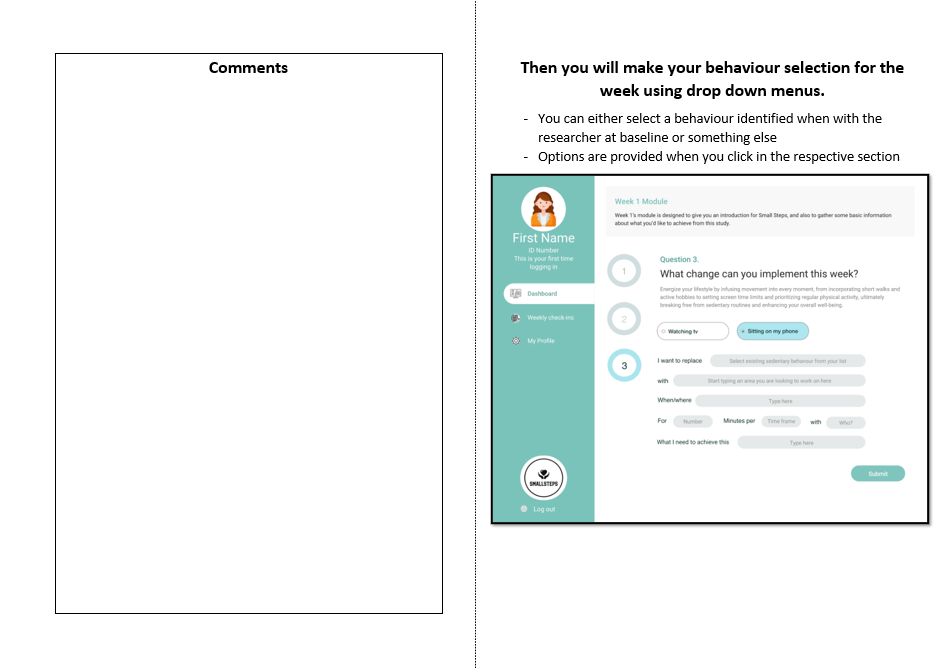
**
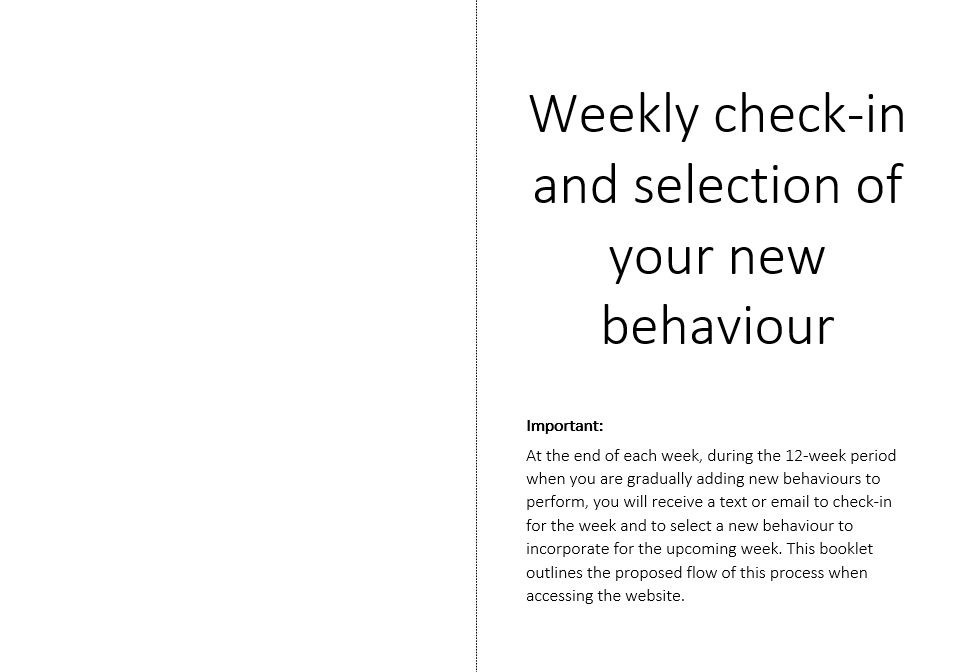
**page 1

page 3

**Supplementary Figure 12.** Workshop 4. Example pages from paper-based workbook of intended website design and action planning process. Participants individually worked through a hard copy booklet of the initial website action planning flow and provided written comments on the opposing page to each respective image regarding any likes and suggestions on the website design and flow of information presented.


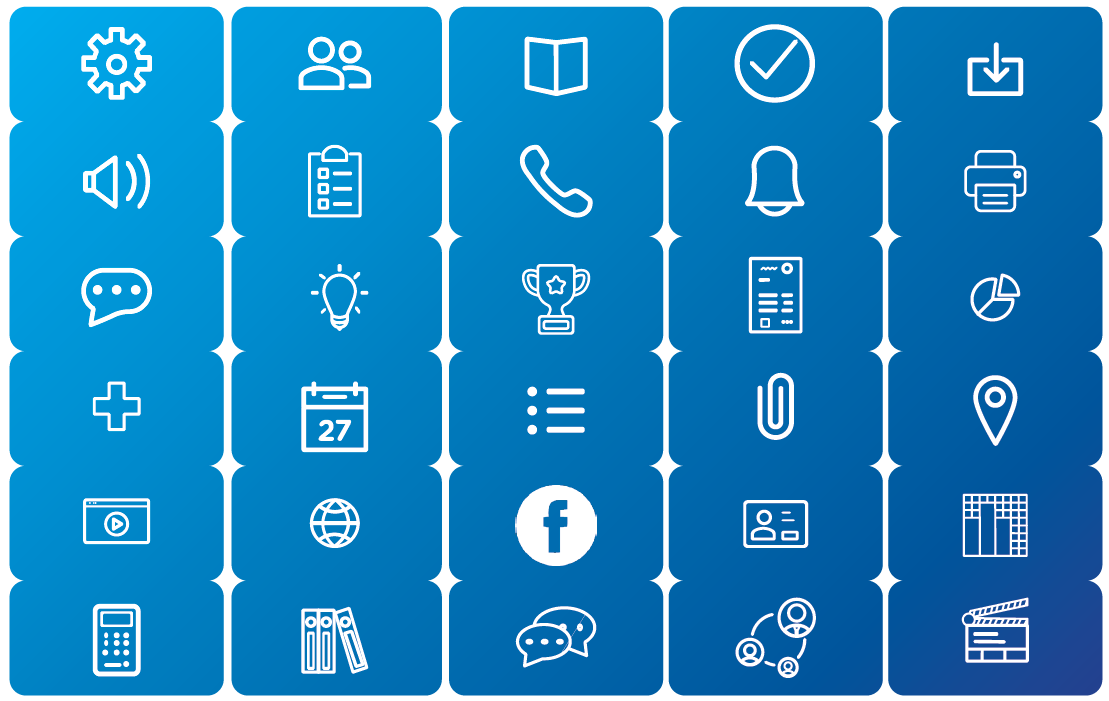

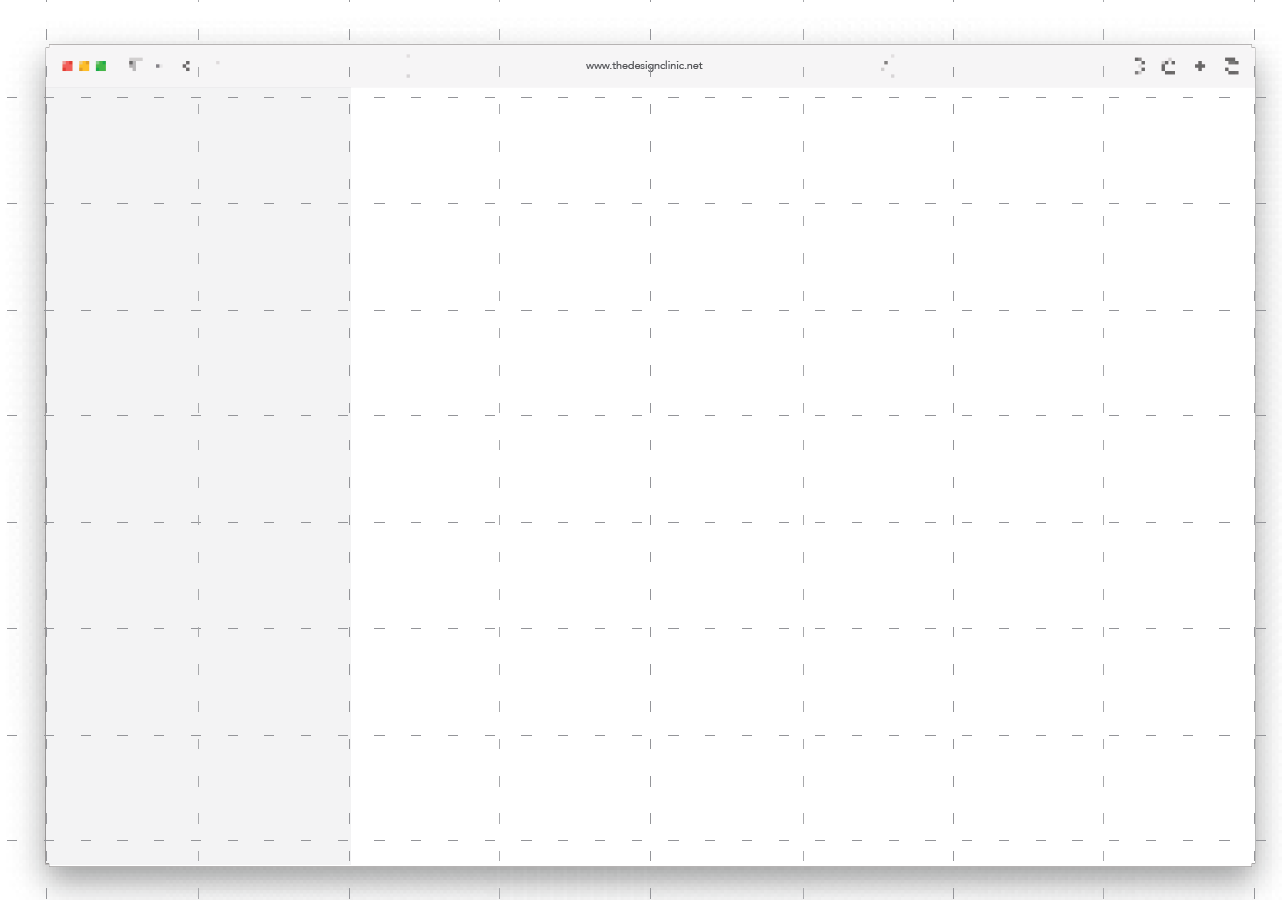
**Supplementary Figure 13.** Workshop 4. Blank template depicting a computer screen (top image) for participants to design their ‘ideal’ intervention dashboard using stickers (bottom image) to promote thinking of desired features.


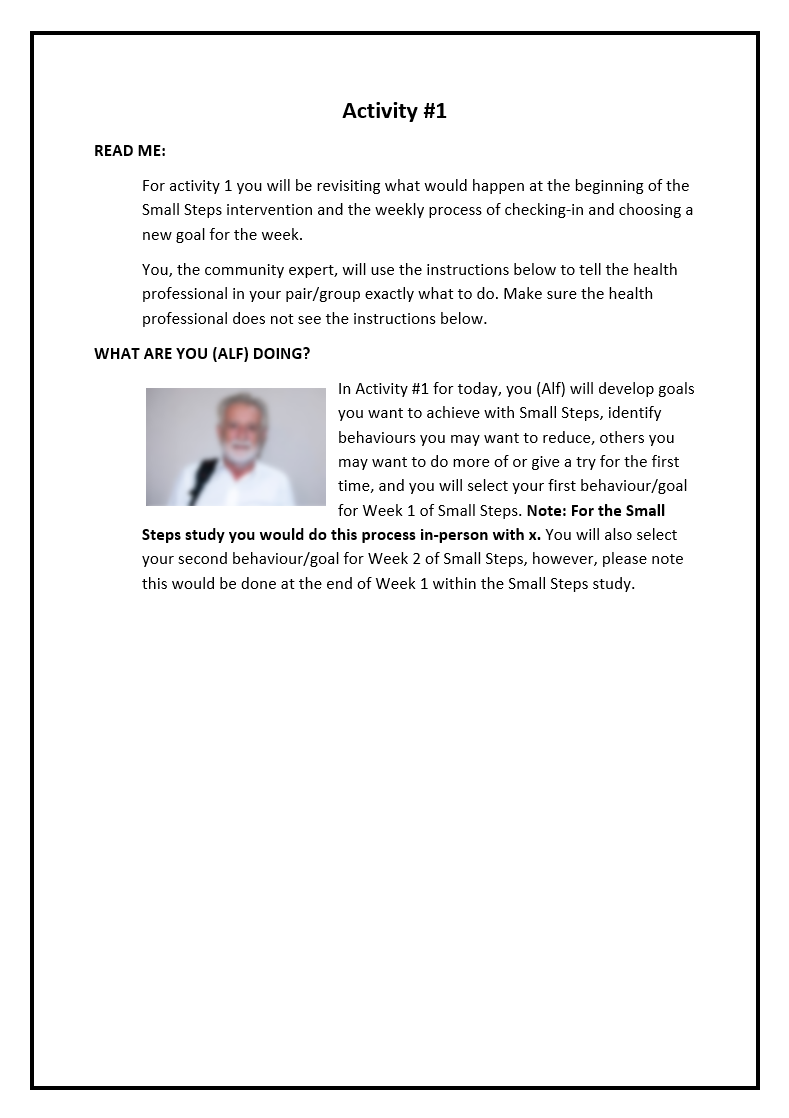

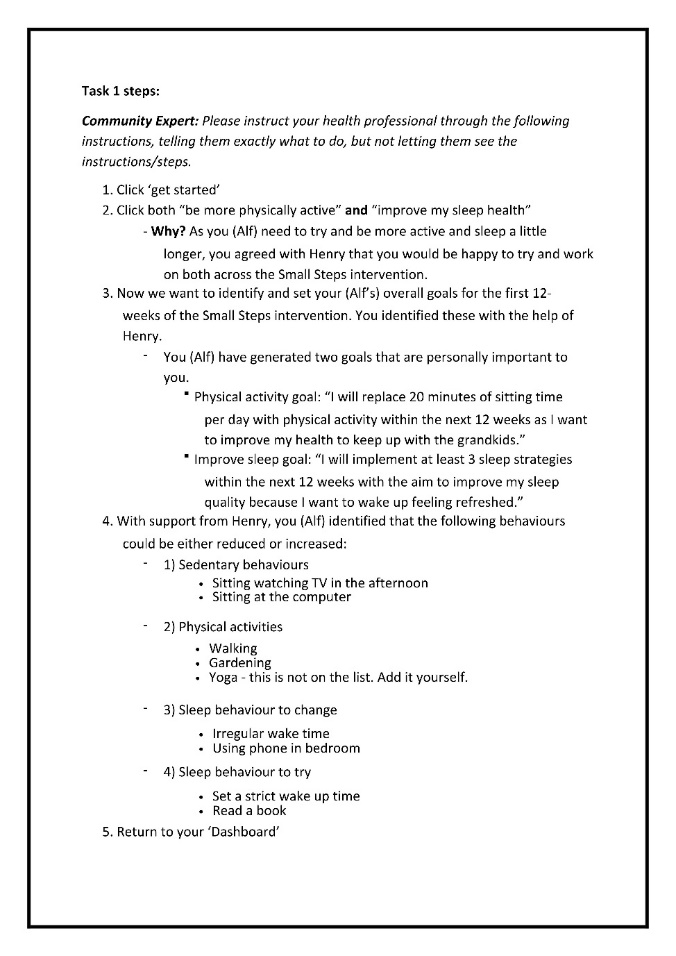


**Supplementary Figure 14.** Workshops 5 and 6. Example of third person role-play scenarios that were developed to allow participants to embody different perspectives (other than their own) and work their way through the intervention website. The level of instruction and prompting decreased throughout each workshop to promote autonomy and determine whether participants were able to learn the website/intervention process promptly or whether removing instruction resulted in confusion.
